# Emerging Native-Similar Neural Representations Underlie Non-Native Speech Category Learning Success

**DOI:** 10.1162/nol_a_00035

**Published:** 2021-06-09

**Authors:** Gangyi Feng, Yu Li, Shen-Mou Hsu, Patrick C. M. Wong, Tai-Li Chou, Bharath Chandrasekaran

**Affiliations:** Department of Linguistics and Modern Languages, The Chinese University of Hong Kong, Shatin, N.T., Hong Kong SAR, China; Brain and Mind Institute, The Chinese University of Hong Kong, Shatin, N.T., Hong Kong SAR, China; Applied Psychology Programme, Beijing Normal University–Hong Kong Baptist University United International College, Zhuhai, Guangdong, China; Imaging Center for Integrated Body, Mind and Culture Research, National Taiwan University, Taipei, Taiwan; Department of Psychology, National Taiwan University, Taipei, Taiwan; Department of Communication Sciences and Disorders, School of Health and Rehabilitation Sciences, University of Pittsburgh, Pittsburgh, PA, USA

**Keywords:** individual differences, non-native speech learning, tone language, predictive modeling, multivariate representation, feedback processing

## Abstract

Learning non-native phonetic categories in adulthood is an exceptionally challenging task, characterized by large interindividual differences in learning speed and outcomes. The neurobiological mechanisms underlying the interindividual differences in the learning efficacy are not fully understood. Here we examine the extent to which training-induced neural representations of non-native Mandarin tone categories in English listeners (*n* = 53) are increasingly similar to those of the native listeners (*n* = 33) who acquired these categories early in infancy. We assess the extent to which the neural similarities in representational structure between non-native learners and native listeners are robust neuromarkers of interindividual differences in learning success. Using intersubject neural representational similarity (IS-NRS) analysis and predictive modeling on two functional magnetic resonance imaging datasets, we examined the neural representational mechanisms underlying speech category learning success. Learners’ neural representations that were significantly similar to the native listeners emerged in brain regions mediating speech perception following training; the extent of the emerging neural similarities with native listeners significantly predicted the learning speed and outcome in learners. The predictive power of IS-NRS outperformed models with other neural representational measures. Furthermore, neural representations underlying successful learning were multidimensional but cost-efficient in nature. The degree of the emergent native-similar neural representations was closely related to the robustness of neural sensitivity to feedback in the frontostriatal network. These findings provide important insights into the experience-dependent representational neuroplasticity underlying successful speech learning in adulthood and could be leveraged in designing individualized feedback-based training paradigms that maximize learning efficacy.

## INTRODUCTION

During infancy, dramatic changes occur in the brain networks that support speech processing (Kuhl, 2004, 2010). Language-general perception narrows to become more selective to the statistical regularities of the native environment (Cheour et al., 1998; Garcia-Lazaro et al., 2011; Kuhl, 2004), promoting greater sensitivity to native speech sound categories (Kuhl, 2010; Nakahara et al., 2004; Vallabha et al., 2007). However, experience-dependent perceptual narrowing can also alter low-level perception and interfere in the acquisition of non-native speech categories in adulthood (Kuhl et al., 2008; Myers, 2014). Non-native speech categories can be acquired to native-like proficiency in adulthood when learners are provided some amount of feedback and with sufficient intensity of training (Lively et al., 1993; Reetzke et al., 2018). However, even in adults with similar language backgrounds, with similar cognitive, socio-economic, motivation, and hearing status, and undergoing identical training paradigms, large interindividual differences define speech learning performance (Ellis, 2004). Indeed, individual differences are ubiquitous in the acquisition of most subcomponents of language (Kidd & Donnelly, 2020; Kidd et al., 2018). This is especially the case when adults with no tonal language experience learn to categorize lexically relevant tone patterns (Chandrasekaran et al., 2010; Wong & Perrachione, 2007). Our goal here is to elucidate the neural mechanisms that underlie the extensive interindividual variability in non-native tone-category learning success. We examine the following questions. First, are the emerging neural representations of linguistic-tone categories in the successful adult learners fundamentally similar or dissimilar to the neural representations that are acquired in early infancy? That is, is the similarity in the neural representations between adult learners and native listeners a robust neural neuromarker of learning success? Second, is the feedback sensitivity in the corticostriatal systems a key indicator of individual differences in learning success and the degree of the putative “nativeness” of neural representations?

These questions relate to theoretical positions adopted in the domain of second language (L2) acquisition to explain individual variability in attainments. Much of the focus in this literature is on the learning of grammatical structures; however, this literature provides a theoretical scaffolding for learning non-native phonology. The shallow structure hypothesis posits that the representations underlying L2 acquisition have less detail relative to those underlying native language (L1) acquisition (Clahsen & Felser, 2006a, 2006b, 2018; also see Ullman, 2006). The fundamental difference hypothesis posits a lack of convergence between non-native and native language representations and proposes that L2 learning necessitates relying on domain-general learning mechanisms, such as executive control functions and feedback processes (Bley-Vroman, 1990, 2009). These theoretical perspectives not only explain the differences between L1 and L2 acquisition and representation, but also imply that the comparison between the representations of non-native learners’ L2 and native speakers’ L1 could potentially reflect the nativeness in L2 processing and attainment for the learners (Birdsong, 2018; Hartshorne et al., 2018). For example, using fMRI with traditional univariate activation-based analysis approaches, quantitative differences and similarities in brain activations have been found between L1 and L2 processes where the degree of similarity is dependent on the level of L2 proficiency and age of acquisition (Abutalebi, 2008; Chee et al., 1999; Feng et al., 2015; Morgan-Short et al., 2015; Perani & Abutalebi, 2005; Perani et al., 1996) Moving beyond the activation-based group-level comparisons between L1 and L2, here we focus on examining the multivariate representation-based neural mechanisms underlying the interindividual variability in the acquisition of a new phonological structure not present in English–lexical tones.

In tone languages, pitch information can alter word meaning (Yip, 2012). For native speakers of tone languages, extracting pitch patterns from the incoming auditory stream and mapping key pitch features to tone categories are critical for speech communication. In contrast, non-native listeners who do not have tonal language experience have great difficulty in discriminating tonal contrasts with similar pitch patterns (Reid et al., 2015; So & Best, 2010). This discrimination difficulty may be mainly because tonal information is not linguistically relevant to non-native listeners’ phonological systems (Best, 1995; Reid et al., 2015). The challenges in discrimination and learning have also been viewed from the perspective of feature-weighting (Chandrasekaran, Gandour, & Krishnan, 2007; Chandrasekaran et al., 2010). For contour-tone languages like Mandarin, at least two pitch-related dimensions define tone categories: pitch height and slope; both native and non-native listeners weight pitch height significantly during categorization. In contrast, the weighting of pitch slope or the combination of pitch height and direction (i.e., contour: time-varying height) is strongly modulated by language experience, with native listeners weighting this dimension more heavily than non-native listeners (Chandrasekaran, Krishnan, & Gandour, 2007). The increasing weighting of pitch contour allows for a more stable mapping of tone categories across contexts and talkers with varying fundamental frequencies like native listeners, which could increase the probability of successful categorization and learning.

Successful learners must establish new representations of novel tone categories by mapping highly time-varying pitch patterns to stable tone categories (Feng et al., 2019), a key step for learning the lexicon. To achieve this, learners would likely need to update their internally emerging representations with an increased weighting of key dimensions that underlie the native tone perceptual space. Here, we assess the extent to which the emerging neural representations of tone categories and the underlying dimensions acquired in adulthood are similar to the representations in native Mandarin listeners who use tones linguistically. We specifically compare the detailed representational structure (including all tonal contrasts under different syllable contexts) in the brain with a hypothesis that more successful learners demonstrate emerging representational patterns that are more similar to those in native listeners.

Finally, we test the hypothesis that the corticostriatal learning systems that are sensitive to feedback valence are critical neural driving sources of individual differences in learning efficacy and the training-related neural representational plasticity in adulthood. An emerging perspective of the neural functionality of feedback is that adult learners require feedback, processed by the corticostriatal networks, to build novel speech category representations (i.e., sound-to-category; Chandrasekaran, Yi, & Maddox, 2014; Feng et al., 2019; Lim et al., 2019; Yi et al., 2016). Per the dual-learning systems (DLS) model (Chandrasekaran, Koslov, & Maddox, 2014; Chandrasekaran, Yi, & Maddox, 2014), a reflective (sound-to-rule mapping) system and a reflexive (sound-to-reward mapping) system operate on a trial-by-trial basis to assist sound-to-category learning. The reflective system involves the frontoparietal attentional network and the hippocampus, which operates by generating and testing hypotheses based on corrective feedback; the reflexive system, on the other hand, involves the striatum in mapping stimuli to motor responses that result in rewards. This DLS model focuses on speech category learning in adulthood. The corticostriatal systems that subserve category learning in the DLS model may be ubiquitous to the acquisition of other language subcomponents for adult learners. Indeed, previous studies have proposed comparable cortical-subcortical systems that drive reward-dependent acquisition of novel words (Ripolles et al., 2014, 2016).

To test the two hypotheses, we analyzed data from a tone-category learning experiment that leveraged previously collected fMRI data to assess emerging representations in English-speaking learners (*n* = 53) as they learned to categorize non-native tone categories with feedback (Feng et al., 2019; Yi et al., 2016). To quantify the degree of the nativeness in neural representational structure for each learner, we conducted a new fMRI study in which a group of native Mandarin speakers (*n* = 33) performed the same tone categorization task with the same set of stimuli as the non-native learners but without feedback. The behavior response patterns of the learners were modeled with representational models that are informed by the acoustic and non-acoustic category-related features as well as the neural representational patterns from the native Mandarin speakers to examine the degree of emerging nativeness in representations. Importantly, using intersubject neural representational similarity (IS-NRS) analysis (Chen et al., 2017; Diedrichsen & Kriegeskorte, 2017), we measured the extent of shared patterns in neural representational structure between learners and native listeners (i.e., IS-NRS). To test our hypotheses, we used a predictive modeling approach (Gabrieli et al., 2015; Rosenberg et al., 2016) with learners’ IS-NRSs as neural predictors to predict their behavioral learning efficacy (i.e., speed and outcome). To further evaluate the predictability of IS-NRS, we compared the predictive power of IS-NRS with those of models with other neural representational measures. To assess the detailed representational structure underlying successful learning, we combined predictive analytics and a data-driven single vector decomposition procedure that estimated the relationship between dimensionality and learning efficacy. Finally, to evaluate the underlying driving factors of the interindividual variability in learning success and emerging representations, we calculated a neural feedback sensitivity index to predict learning speed and outcome as well as the degree of learners’ nativeness in neural representations (i.e., IS-NRS).

## MATERIALS AND METHODS

### Participants

Native speakers of Mandarin (*n* = 33; 18 females; right-handed; age range, 20–37 years; mean age = 25.5 years) were recruited from the communities around the National Taiwan University, Taipei. These native participants were highly proficient at listening and speaking in standard Mandarin. They were recruited to participate in a tone categorization fMRI experiment designed specifically for the current study. This experiment was approved by the Research Ethics Committee at National Taiwan University. Native speakers of English were recruited from the communities around The University of Texas at Austin (*n* = 53; 39 females; right-handed; age range, 18–35 years; mean age = 21.8 years). These English-speaking participants did not have tonal language experience and had minimal formal music training experience (<3 years). All the participants reported normal hearing ability, which was confirmed by audiological testing (pure tone thresholds < 25 dB HL at 1, 2, and 4 kHz). They had normal or corrected-to-normal vision and did not have any neurological impairments. Training protocols and materials were approved by the Institutional Review Board of The University of Texas at Austin. All participants provided written informed consent and were monetarily compensated for their time.

### Stimulus

Natural exemplars (*n* = 40) of the four Mandarin tones (T1: high-flat; T2: low-rising; T3: low-dipping; T4: high-falling) were generated by two native Mandarin speakers (originally from Beijing; 1 female) in the context of five monosyllabic Mandarin Chinese words (/bu/, /di/, /lu/, /ma/, and /mi/). (See spectrograms of sample stimuli in Figure S1A in the Supplementary Materials. Supporting Information can be found online at https://www.mitpressjournals.org/doi/suppl/10.1162/nol_a_00035). These syllables were chosen because they also exist in the American English syllabic inventory. Therefore, the neural representations of native and non-native speech categories could be examined for the learners and compared with the native Mandarin speakers. The stimuli were normalized for a root mean square (RMS) amplitude of 70 dB and a duration of 442 ms (Perrachione et al., 2011). Both learners and native Mandarin speakers heard the same set of stimuli during the experiments.

### Experimental Procedure

In the native tone-categorization fMRI experiment, Mandarin-speaking participants were required to categorize sounds into one of the four categories during scanning by pressing the “1”, “2”, “3”, or “4” buttons using an in-scanner response box, with category-response mapping counterbalanced across participants. Native participants were not provided feedback following categorization responses. They briefly practiced categorization before scanning to establish category-response mapping. To reduce the interference of scanner noise to speech perception, we employed a customized sparse-sampling imaging sequence with an 800-ms silence gap between every two consecutive imaging acquisitions (Figure S1B). Each sound was presented (duration = 442 ms) within the silence gap 100 ms after each imaging acquisition. Each sound was presented once in each block, and the order of stimuli randomly varied across blocks. To better estimate the hemodynamic responses for each trial, we added 20 null trials (i.e., silence, duration = 5 s) randomly between sound trials as jittered intertrial intervals in each block (i.e., scan run). To accurately estimate the activation patterns of the sounds, native participants completed at least five blocks of 40 trials each of tone categorization (six participants completed five blocks (200 trials), and 27 completed six blocks (240 trials). Each sound (e.g., /bu4/; collapsed across talkers) was repeated 10 to 12 times. The significant number of repetitions for the same item ensures a sufficient signal-to-noise ratio and accurate activation estimation.

**Figure 1.**
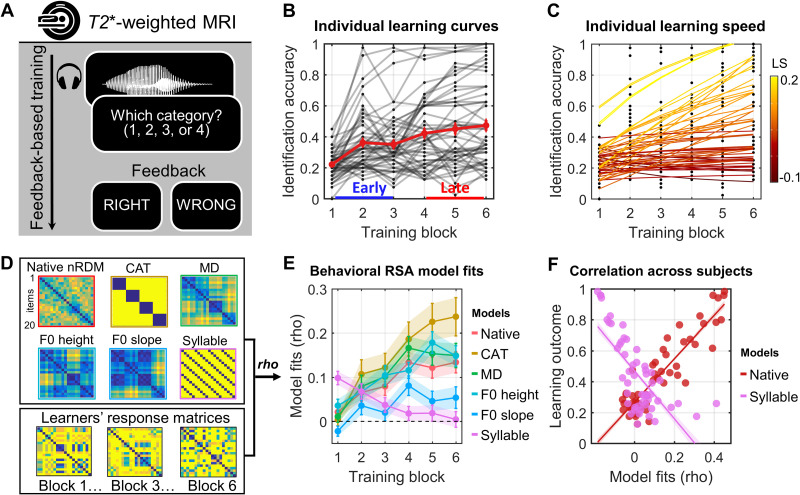
Behavioral tone-category training procedure, learning performance, and response-pattern modeling. (A) Feedback-based sound-to-category training procedure was used during MRI scanning for the learners. The native Mandarin listeners performed the same tone categorization task but without feedback (see Figure S1 for the experimental procedure). (B) Line graphs show the group-level and individual learning curves across six training blocks. Early, the early phase of training; Late, the late phase. (C) Learning speed (LS) was estimated by fitting each learner’s block-by-block accuracies with a power function. See Materials and Methods for the detailed learning curve modeling procedure. (D) Six predefined representational dissimilarity matrices (RDMs) were constructed to model learners’ categorization response patterns using the behavioral representational similarity analysis (bRSA): Native nRDM = native listeners’ neural RDM derived from a predefined brain mask; CAT = binary tone-category RDM; MD = multidimensional pitch RDM; F0 height = pitch height RDM; F0 slope = pitch direction RDM; Syllable = binary syllable-identity RDM; See Materials and Methods for the detailed RDM construction procedure. (E) The bRSA reveals that native-similar tone-category-related information emerges following training, whereas task-unrelated segmental information decreases. Error bar: *SEM* (Standard Error of the Mean). (F) The model fits of the native nRDM (also other tone-category-related RDMs) are highly correlated with the learning outcome (red dots) and speed (not shown). In contrast, an inverse relationship was found between the Syllable model fits and learning outcome (pink dots).

The non-native sound-to-category training procedure has been extensively described in previous studies (Feng et al., 2019; Yi et al., 2016). Briefly, English-speaking participants performed a tone categorization task during scanning, in which they were required to learn to map the sounds onto four categories. The fMRI experiment consisted of six contiguous training blocks of 40 trials each. In each block, each trial started with a fixation cross, and the auditory stimulus was presented for 442 ms. Participants were required to make a categorization response within 2 s. Following the stimulus presentation and categorization response, corrective feedback (i.e., “RIGHT” or “WRONG”) was displayed for 750 ms (see Figure 1A). If the participant did not respond within the 2 s, the response did not record and warning feedback was presented (i.e., “TIME”). To effectively model brain signals for stimulus and feedback presentation separately, we employed a jittered stimulus-feedback interval design (2–4 s; feedback-stimulus interval = 1–3 s; pooled from a uniform distribution) (Birn et al., 2002; Dale, 1999; T. T. Liu et al., 2001). Each sound stimulus was presented once within each block, with a total of 240 trials in the training experiment.

### Imaging Acquisition

For the native tone-categorization experiment, all MRI data were acquired using a Siemens 3T Magnetom Prisma MRI system with a 20-channel head coil at Imaging Center for Integrated Body, Mind, and Culture Research, National Taiwan University. Functional images were acquired using a T2*-weighted gradient echo-planar imaging (EPI) pulse sequence (repetition time [TR] = 2,500 ms with 800–ms silence gap; echo time [TE] = 30 ms; flip angle = 90°; 31 slices; field of view [FOV] = 224 × 224 mm^2^; in-plane resolution = 3.5 × 3.5 mm^2^; slice thickness = 3.5 mm with 1.1 mm gap; acceleration factor = 2). T1-weighted high-resolution structural images were acquired using a magnetization prepared rapid acquisition gradient echo (MPRAGE) sequence (192 slices; TR = 2.0 s; TE = 2.3 ms; flip angle = 8°; voxel size = 0.94 × 0.94 × 1 mm^3^).

For the sound-to-category training experiment, MRI data were acquired using a Siemens 3T Magnetom Skyra MRI system with a 32-channel head coil at the Biomedical Imaging Center at The University of Texas at Austin. Functional images were obtained using a gradient-echo multi-band EPI pulse sequence (flip angle = 60°; TR = 1.8 s; TE = 30 ms; FOV = 250 × 250 mm^2^; in-plane resolution = 2 × 2 mm^2^; 36 axial slices; slice thickness = 2 mm; distance factor = 50%) using GRAPPA with an acceleration factor of 2. Whole-brain T1-weighted structural images were obtained via MPRAGE sequence (176 slices; TR = 2.53 s; TE = 3.37 ms; FOV = 250 × 250 mm^2^; 256 × 256 matrix; voxel size = 1 × 1 × 1 mm^3^; distance factor = 0%).

### Behavioral Data Modeling

#### Estimation of learning outcome and speed

The learning outcome is defined as the average tone identification accuracy in the last three blocks. There are three considerations for this learning outcome definition. First, at the group level, learning performance in the last three blocks was relatively stable compared to the first three blocks. That is, tone identification performance was not significantly improved within the last three blocks (*p*’s > 0.05), which suggests that the last three blocks may be a relatively stable learning phase. Second, individual differences in learning outcomes are based on the fact that the amount of training was the same across learners. Therefore, we selected the same number of training blocks for each learner. Third, the division of two training phases (i.e., the first and the last three blocks) ensures that there are enough trials for the brain estimation of stimulus items for the multivariate analyses. Based on the above considerations, the last three blocks were defined as the “late phase” of training, and the first three blocks were defined as the “early phase.” It is worth noting that this training phase definition mainly refers to the amount of training that the learners received instead of the proficiency level in a certain phase achieved by individual learners.

To model the non-native tone learners’ learning curves properly for the estimation of learning speed, we used four functions (i.e., hyperbolic, logarithmic, power, and linear regression functions) to fit each subject’s block-by-block category identification accuracies, separately (Figure 1B & Figure S2). The goodness of fit (GOF) of the curve modeling of each function was first calculated by estimating the RMS error between a fitting line and the actual learning curve for each learner. The resulting GOF of the three curvilinear functions was then compared with that of the linear function at the group level to examine whether the curvilinear functions were better in capturing the learning progress than the linear function. We found that only the GOF of the power function (i.e., *Y* = *aX* ^*b*^; *X* = training block and *Y* = tone identification accuracy) was significantly better than the linear function (*t*(52) = −4.16, *p* < 0.001; lower is better in curve fitting). Parameters *a* and *b* from the power function are both associated with the learning progress. The parameter *a* represents the steepness of a learning curve, denoting the initial learning gain based on the same amount of training or a “conversion factor” between the amount of training and learning performance. The parameter *b* represents the changes in learning gain across different training blocks. Both parameters contribute to the construct of learning speed (see individual fitting curves with the power function in Figure S2). Therefore, we combined the two parameters by multiplying them to represent the learning speed (i.e., LS = *a* × *b*; see Figure 1C). The learning speed was significantly correlated with the learning outcome (*r* = 0.91, *p* < 0.001) but it was not significantly correlated with the first block categorization performance (*r* = 0.216, *p* = 0.120). The two learning measures (i.e., learning outcome and speed) share 82% variances. That is, there are around 18% of nonoverlapped variances that are unique to each learning measure. We hypothesize that these nonoverlapped variances may be predicted by different neural sources. Therefore, we used both measures as learning success indices for predictive modeling.

#### Categorization response-pattern modeling with behavioral representational similarity analysis

We estimated learners’ behavioral representational structure during training by using behavioral representational similarity analysis (bRSA) to model their behavioral response confusion patterns (Figure 1D, bottom panel). The bRSA reveals the model fits (i.e., Spearman’s correlations) between predefined representational models (i.e., representational dissimilarity matrices [RDMs]) and the response confusion matrices for each block. We created six RDMs to examine what type of dimensions/information emerges or changes following training. These RDMs are 20-by-20 dissimilarity matrices with four tones and five syllables, including dissimilarities between all pairs of tonal contrasts. The dissimilarities were calculated based on different acoustic and non-acoustic information, including native neural activation patterns (Native nRDM), binary tone-category labels (CAT), fundamental frequency (F0) height, F0 slope, and syllable identity (see Figure 1D, upper panel).

The native nRDM was constructed based on the neural activation-pattern dissimilarities between each pair of sound items derived from the native Mandarin speakers within a predefined speech/auditory perception-related brain mask (see Figure S3). This mask was generated from a meta-analysis with Neurosynth (https://neurosynth.org/) by searching the topic dataset with keywords “speech,” “auditory,” and “perception.” The dataset consists of 400 topics extracted with linear discriminant analysis (LDA) from the abstracts of all articles in the Neurosynth database as of July 2018. This automatic meta-analysis included 269 studies (Topic 180) with a list of highly related topic words, including “auditory,” “perception,” “speech,” “non-speech,” “sound,” “processing,” and so on.

We used this independent brain mask to avoid any ROI-selection bias. This brain mask was only used for creating native nRDM for bRSA. The purpose of including this native nRDM model for the bRSA was to estimate to what extent the learners’ behavioral response patterns were similar to the native neural representation patterns. The F0 height RDM was constructed by calculating the acoustic distance between each pair of sounds based on their mean F0 estimates. The F0 slope RDM was constructed by calculating the distance between each pair of sounds based on their F0 slopes (i.e., F0 height changes over time). For the multidimensional (MD) model, we created a two-dimensional space with the F0 height and slope dimensions. The Euclid distance between each sound pair within this two-dimensional space was computed and converted into a distance matrix. Each dimension was normalized before calculating the distance. (See Feng, Gan et al., 2018, for the detailed RDM construction procedure.) We then normalized these RDMs by scaling between 0 (low dissimilarity, i.e., close in distance) and 1 (high dissimilarity, i.e., far from each other in distance). The binary tone-category RDM was constructed based on combinations of the four category labels (i.e., 0 for the same category, 1 for different categories). The syllable RDM was constructed based on the identity of the five syllables (i.e., 0 for the same syllable, 1 for different syllables). These six RDMs were correlated with learners’ response confusion matrices in a block-by-block manner. Learners’ response confusion matrices were created based on their categorization responses. If two sounds had an identical response, then this pair was coded as 0 in the confusion matrix; otherwise, it was coded as 1. Using this procedure, we created two confusion matrices in each block (one for each talker) for each learner. The two matrices were then averaged for each block. Finally, we calculated the Spearman’s correlations (i.e., model fits) between each RDM and confusion matrices. We also examined the relationships between the RDM model fits and learning outcome and speed across subjects to see which RDM explains most of the interindividual variance in learning success.

### Neuroimaging Data Analysis

#### Preprocessing for multivariate pattern analyses

All MRI data were preprocessed using SPM12 (Wellcome Department of Imaging Neuroscience, London, UK; www.fil.ion.ucl.ac.uk/spm/). Briefly, functional images were head-movement corrected by coregistering each image with the mean image. The high-resolution structure image was coregistered to the mean functional image for each subject. The normalization transformation parameters were then estimated using a segmentation-normalization procedure with the coregistered structure image. The normalization parameters were used to normalize the functional images to the Montreal Neurological Institute (MNI) space for group-level statistical analyses. To model single-trial brain activation responses for multivariate pattern analysis (MVPA), the realigned functional images in the native space were fed into the subject-level generalized linear model (GLM) analysis with the least-squares single approach (Mumford et al., 2012, 2014). Specifically, for the tone-category training dataset, a design matrix was constructed with a regressor of interest for each trial during sound or feedback presentation; a regressor of noninterest consisted of other events (i.e., feedback or sound presentation for the current trial, and stimulus and feedback presentations for the other trials), six head movement regressors, and a session mean regressor for each training block individually. Therefore, 480 subject-level GLM models (240 models for sound presentation and another 240 models for feedback) were constructed and estimated for each subject for the training experiment. Similarly, for the native tone-categorization experiment, 200 or 240 subject-level GLM models (for the sound presentation events) were constructed and estimated. The *t* statistic brain maps were calculated for each trial and further used for MVPA (Misaki et al., 2010).

### Multivariate Pattern Analyses

We calculated three types of MVPA measures for learning-success prediction, including IS-NRS, model-based representational similarity analysis (RSA) measures, and neural feedback sensitivity. The three types of measures were considered predictive features for predictive modeling (see Figure S4A for the overview of the data processing pipeline).

#### IS-NRS analysis

**Figure 2.**
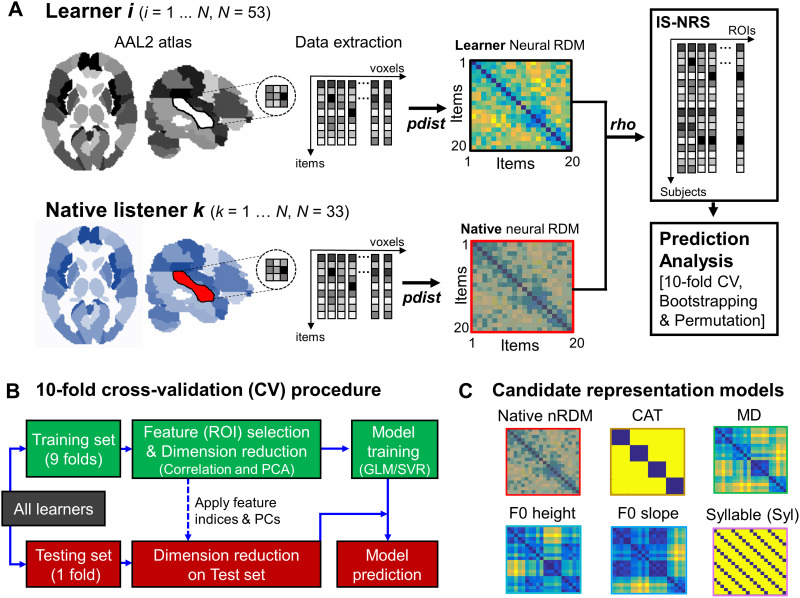
Intersubject neural representational similarity (IS-NRS) analysis, predictive modeling procedure, and candidate representation models for RSA and prediction analysis. (A) Graphical illustration of the calculation of IS-NRS. Neural activation patterns were extracted from predefined ROIs based on the AAL2 atlas for both learners and native listeners. The neural RDMs (nRDMs) were calculated separately for each group. Each learner’s nRDM was compared with every native listener’s nRDM for each ROI. The IS-NRSs were then generated and used as predictive features to predict learning success (i.e., outcome and speed). (B) The 10-fold cross-validation (CV) procedure for model construction and validation. All learners were split into 10 folds where 90% of the learners’ data were used to train a GLM/SVR model and then the trained model was used to predict the left-over 10% of the learners. This procedure was repeated 10 times. Predictive powers were estimated by calculating correlations between the predicted and observed learning performance. Permutation and bootstrapping procedures were used to determine the statistical significance and stability of the predictive powers. See Figure S4 for the overall data analysis pipeline. (C) Candidate representational models for the generation of neural representational predictive features. To compare the predictive powers of IS-NRS (i.e., derived from the ROI-based native nRDM) with those of other model-based representational measures, the same prediction analyses were conducted with the predictors derived from the other five RDMs (i.e., CAT, MD, F0 height, F0 slope, and Syl).

We quantified the degree of nativeness in neural representational structure for the learners by measuring the IS-NRS between each non-native learner and each of the native-Mandarin speakers for each anatomical defined region (see Figure 2A for a graphical illustration of the analysis procedure). Higher IS-NRS indicates greater similarity in the neural representations of the speech sounds relative to the native listeners. The IS-NRS analysis is a derivative of the RSA enabling us to evaluate similarity in neural representational structures (i.e., nRDMs) between subjects within the same stimulus space instead of in the subjects’ voxel space (Kriegeskorte et al., 2008; Kriegeskorte & Kievit, 2013). In the IS-NRS calculation pipeline, nRDMs were first generated within each subject and compared between subjects from the two groups (Figure 2A). Since both groups of participants heard the same sets of sounds, their neural representations were comparable in the same space. This two-step dissimilarity-similarity analysis approach can capture the similarity in representational structure between subjects and datasets from different scanners, populations, imaging modalities, and even species while ensuring that the representational similarity effect is not due to the differences between datasets in these variables.

To calculate the IS-NRS, we extracted activation patterns from 94 regions of interest (ROIs) for both the learners and the native listeners based on an anatomical-defined atlas, Anatomical Automatic Labeling 2 (AAL2; Rolls et al., 2015). Cerebellum regions were removed because the cerebellum was not entirely covered for some of the learners’ data. Since two groups of datasets differ slightly in imaging parameters (e.g., number of voxels and voxel size) and there are large interindividual differences in brain anatomy, the ROI-based approach ensures that the neural representational structures were compared in the same anatomical-defined areas between the two groups of subjects. The AAL2 atlas in the MNI space was projected back to the native space for each subject, and the activation patterns when listening to the stimuli were extracted for each ROI (Figure 2A). Then, the nRDMs were calculated based on the activation patterns for each ROI and subject (note that dimensionality reduction was additionally conducted before the nRDM calculation to evaluate the representational dimensionality underlying successful learning; refer to the Representational Dimensionality Evaluation section). Calculating nRDMs ensures that different subjects’ neural patterns were converted onto the same stimulus representational space. In this space, the distance (i.e., dissimilarity) between each pair of sounds (or phonetic contrasts) can be quantified based on their activation patterns and can be compared with other subjects or model-based RDMs. For each ROI, we calculated the IS-NRS for each learner-native speaker pair with Spearman’s ranking correlation based on two vectorized nRDMs. The variances of hand-response RDM (left vs. right hand) were controlled for using the partial correlation approach to rule out the potential confounding of hand-response pattern similarity between the two groups. Therefore, each learner has 33 IS-NRSs (33 native listeners) for each ROI. These IS-NRSs were then averaged for the same learner in each ROI. The resulting IS-NRS data (i.e., a learner-by-ROI matrix) were used as predictive features to train and validate prediction models for the prediction of individual learners’ behavioral learning outcome and speed.

#### Representational dimensionality evaluation

To evaluate the representational dimensionality underlying successful learning (i.e., how many dimensions underlying learners’ representational structure explain most individual differences in learning success), we additionally used principal component analysis (PCA) with the single vector decomposition algorithm to decompose learners’ sound-induced activation patterns into principal components (PCs) before IS-NRS calculation. We used a different number of PCs (maximal 20 PCs due to 20 sound items) to re-calculate the distance (i.e., Euclidean distance) between each sound pair and to construct the nRDMs for the learners. The IS-NRSs were calculated based on the dimension-constrained nRDM (see the procedure in Figure 5A and the detailed processing pipeline in Figure S4). Finally, a subject-by-ROI-by-PC IS-NRS matrix was obtained for the predictive modeling analysis. By using this dimension decomposition approach with predictive modeling, we can assess the dimensionality underlying individual learning success and parametrically examine the relationship between representational dimensionality and individual differences in learning success.

#### Model-based RSA and searchlight approach

To compare the predictive power of the IS-NRS and other representational measures, we calculated the representational similarities between learners’ nRDMs and five predefined model-based RDMs. The five RDMs were derived based on the acoustic properties and phonetic category labels of the stimuli (i.e., CAT, MD, F0 height, F0 slope, and Syllable; see Figure 2C). These model-based representational similarities were then entered into the predictive modeling to estimate to what extent these representational measures could be predictors of learning success. We further examined whether the predictive power of IS-NRS outperforms these model-based RSA measures. Cross-validation with bootstrapping and permutation procedures was used to determine the statistical significance and stability of the predictive models (see the next section for details).

We also conducted model-based RSA with the searchlight approach (Kriegeskorte et al., 2006) for the five model RDMs to examine how the learners’ neural representations of the stimuli-related information change following training. The same searchlight RSA was also conducted for native speakers for comparison. This searchlight approach has been described extensively in previous studies (Feng, Gan et al., 2018; Feng et al., 2019, 2021). We briefly described the approach here. This searchlight RSA analysis was conducted across the whole brain. In each searchlight sphere (radius = 3 voxels), an nRDM was generated and then correlated with each of the five model RDMs with Spearman’s correlation. The correlation value was then normalized with the Fisher *z*-Transformation. This *z* value was then mapped back to the center voxel of the sphere. This RSA was conducted for each voxel to generate representational maps for each learner. We conducted this searchlight RSA for the early and late blocks separately. For the group-level analysis, the individual RSA maps in the learners’ native space were first normalized to standard MNI space and then fed to a one-sample *t* test against chance.

#### Neural sensitivity to feedback valence

To examine the extent to which individual differences in neural sensitivity to corrective feedback relates to learning success and learners’ nativeness in neural representations (i.e., IS-NRS), we estimated the ROI-based feedback-type classification accuracies and used them as predictors to predict individual learning outcomes and speed as well as the IS-NRS. We operationally defined neural sensitivity to feedback valence as the feedback-type classification accuracy (correct vs. incorrect feedback) based on the single-trial brain activation patterns. In each ROI, we used an LDA classifier (Chang & Lin, 2011) with a leave-one-block-out cross-validation (CV) procedure to classify individual trials’ feedback types (correct or incorrect). Missing trials (i.e., trials for which there was no response; 7.1% on average across non-native learners) were removed before the classification analysis. We conducted the classification analysis separately for the early and late phases of training. Two learners were removed from the analysis because they achieved 100% accuracy in one of the last three blocks. The ratio of correct and incorrect feedback trials was varied across learners and training phases. To avoid this inherent imbalance, we used a balanced leave-one-block-out partition procedure. This procedure randomly selected the same number of correct and incorrect feedback trials for both training and testing so that each feedback type occurs equally often in the training and testing chunks in the CV procedure. Higher classification accuracy indicates higher neural sensitivity to feedback-valence. The ROI-based classification accuracies were used as predictive features to predict learning outcomes and speed as well as the IS-NRS. If the predictive power is significantly higher than chance, the neural feedback sensitivity plays a critical role in learning success and the emergence of native-similar neural representations.

### Predictive Modeling Analysis

To determine whether the neural measures significantly predict learning outcomes and speed as well as the nativeness of the emerging neural representations, we used multiple linear regression and linear support vector regression (SVR) as prediction algorithms in combination with a 10-fold CV procedure to train and validate prediction models. The neural measures (i.e., IS-NRS, five model-based representational measures, and neural feedback sensitivity index) obtained from all ROIs were used as predictive features, separately. Neural measures from all subjects were combined into an *S*-by-*F* matrix where S is the number of subjects, and F is the number of features (i.e., ROI).

We used a nested 10-fold CV procedure for feature selection, dimension reduction, model construction, and estimation (see Figure 2B and Figure S4B for graphical illustrations). This CV procedure avoids obtaining overfit models with a large number of noisy features and ensures testing the models with unseen data points (Feng, Ingvalson et al., 2018). The nested CV procedure consisted of two levels of nesting (inner and outer) for feature selection, dimension reduction, and model validation. At the inner level, we employed the linear Pearson correlation analysis to remove irrelevant features based on the training sets (Pereira et al., 2009; Smialowski et al., 2010), where only features (e.g., IS-NRS in the superior temporal gyrus [STG]) showing significant correlations with learning outcomes or speed were selected. To avoid selecting features that were related to learners’ first block tone categorization performance instead of speech category learning success (i.e., speed and outcome), we controlled for the interindividual variance of the categorization accuracy in the first block in the feature selection step. Therefore, the predictive powers of the models reflect how well those selected ROIs predict learners’ learning efficacy.

Different feature selection thresholds (i.e., *p* = 0.01 and 10% of total features) were tested to assess the consistency and stability of the predictive performance. To further reduce the dimensionality of the predictors, we conducted the PCA for the selected features and selected the relevant PCs (*p* < 0.05) for further model training. The feature selection and dimension reduction procedures were conducted only on the training set, which was independent of the outer-level model testing (Figure 2B). That is, 90% (i.e., 9-fold) of the data were used for feature selection, dimension reduction, and model training while the hold-out 10% were for testing, repeating 10 times (i.e., 10-fold CV). The linear SVR algorithm with default parameters (i.e., C = 1, Gamma = 1/number of features) was also used to access the multivariate predictive power of the predictors. We used functions from the MATLAB package LIBSVM (Chang & Lin, 2011) in combination with in-house scripts to conduct the predictive modeling analysis. We examined the predictive power of a given neural measure by calculating the Pearson’s correlation between the predicted and observed scores (*rval*_[predicted,observed]_). The predictive modeling analysis was conducted separately for the early and late phases of training.

The statistical significance of the prediction was evaluated using a non-parametric permutation procedure. To test whether the predictive power of each model occurred by chance, we used a non-parametric permutation procedure to generate a null distribution of the predictive power by fully shuffling the predictive features and learning performance across learners for each CV. Note that each feature and learning performance was permuted independently to generate a fully randomized data matrix, and the 10-fold CV procedure was conducted based on the randomized dataset. This data randomization and CV procedure were repeated 10,000 times, and the 95th percentile points of each distribution were used as the critical values for a one-tailed *t* test against the null hypothesis with *p* = 0.05. To test the stability of the prediction, we used a bootstrapping procedure by randomly dividing all the learners into ten folds and conducted the 10-fold CV. Each CV prediction would be slightly different because the composition of the training and testing subjects were different for each iteration. We repeated this bootstrapping procedure 10,000 times. We identified the most contributing regions by comparing each region’s correlation values derived from the feature selection procedure with its corresponding permutation-based correlation distribution. These regional permutation-based *p* values were corrected with the false discovery rate (FDR) approach.

## RESULTS

### Behavioral Results

Tone categorization performance for the native Mandarin speakers was close to ceiling (accuracy = 97.3 ± 2.66 % [mean ± *SD*], reaction time [RT] = 927.98 ± 109.26 ms). In the tone-category training fMRI experiment, English-speaking participants learned to categorize Mandarin tones significantly above chance following training (first block: the mean accuracy across the participants was 22%, range = 0–45%, *SD* = 9%; chance level = 25%; first block vs. chance: *t*(52) = −2.38, *p* = 0.021; the final block: the mean accuracy was 47%, range = 13–100%, *SD* = 26%; final block vs. chance: *t*(52) = 6.27, *p* < 0.001; see Figure 1B for the group and individual learning curves). The category identification accuracy significantly increased over blocks (the first vs. final block paired *t* test: *t*(52) = 7.69, *p* < 0.001). Similarly, the mean accuracy of the late phase of training (i.e., the last three blocks) was significantly higher than in the early phase (*t*(52) = 6.41, *p* < 0.001).

The learning outcome was operationally defined as the mean accuracy in the late training phase. Learning speed was operationally defined as the model fitting parameters for individuals’ learning curves with a power function (Figure 1C). Learning speed was not significantly correlated with the accuracy in the first block (*r* = 0.216, *p* = 0.12) whereas learning outcome was significantly correlated with the accuracy in the first block (*r* = 0.45, *p* < 0.001). These results demonstrate that compared to the outcome, learning speed may be more related to learners’ sound-to-category learning gains instead of the first block accuracy. Because the learning speed and outcome are two target indices reflecting learning efficacy, we used both for the predictive modeling analyses while controlling for the interindividual variance of block 1 accuracy.

The bRSA (see Figure 1D for graphical analysis procedure) showed that RSA model fits significantly increased over blocks for the native nRDM (repeated measures ANOVA; main effect of block: *F*(5, 260) = 10.42, *p* < 0.001) and other category-related RDMs, including CAT (*F*(5, 260) = 20.24, *p* < 0.001), MD (*F*(5, 260) = 12.78, *p* < 0.001), F0 height (*F*(5, 260) = 13.43, *p* < 0.001), and F0 slope (*F*(5, 260) = 5.94, *p* < 0.001). However, the RSA model fits of the Syllable RDM decreased over blocks (*F*(5, 260) = 9.24, *p* < 0.001; Figure 1E). Moreover, individual differences of the model fits were significantly correlated with the individual differences of learning outcome (Native nRDM: *r* = 0.91; CAT: *r* = 0.95; F0 height: *r* = 0.81; F0 slope: *r* = 0.83; MD: *r* = 0.89; Syllable: *r* = −0.73; *p*’s < 0.001; see Figure 1F for a representative scatter plot) and speed (Native nRDM: *r* = 0.80; CAT: *r* = 0.85; F0 height: *r* = 0.72; F0 slope: *r* = 0.74; MD: *r* = 0.78; Syllable: *r* = −0.61; *p*’s < 0.001). These modeling results indicated that native-similar categorization response patterns emerged for the learners following training. The response patterns were highly related to the individual differences in pitch encoding and learning success.

### The Degree of Nativeness in Neural Representational Structure Predicts Learning Success

We employed IS-NRS analysis to measure the degree of nativeness in neural representational structure (i.e., IS-NRS) for individual learners, as compared to a group of native Mandarin-speaking listeners (see Figure 2 for the IS-NRS calculation procedure). Significant similarities with native listeners in neural representational structure emerged at the late phase of training in the bilateral STG and right precentral gyrus (R.PreCG) (Figure 4B). Similar to the emerging native-similar neural representations, the learner’s neural representations of tone categories and pitch-related information emerged in the late phase of training, demonstrated using the searchlight-based RSA with predefined category and pitch-related RDMs (CAT, MD, F0 height, and slope RDMs; see Figure S5; also see Figure S6 for IS-NRS comparisons between learners and native speakers).

Comparing the whole-brain searchlight model-based RSA brain maps between the native listeners and the learners for the tone-category-related RDMs, we found that the searchlight RSA patterns of the learners in the late phase were approaching the patterns of the native listeners, although the RSA correlations were less robust in extent and yielded lower intensity. In contrast, the syllable-related information was less and less represented in the brain following training (Figure S5). Altogether, these results indicate an increase in the learners’ neural representations of learning- or task-relevant tone-category-related information, but a decrease in their representations of learning- or task-irrelevant segmental units (e.g., consonants and vowels).

**Figure 3.**
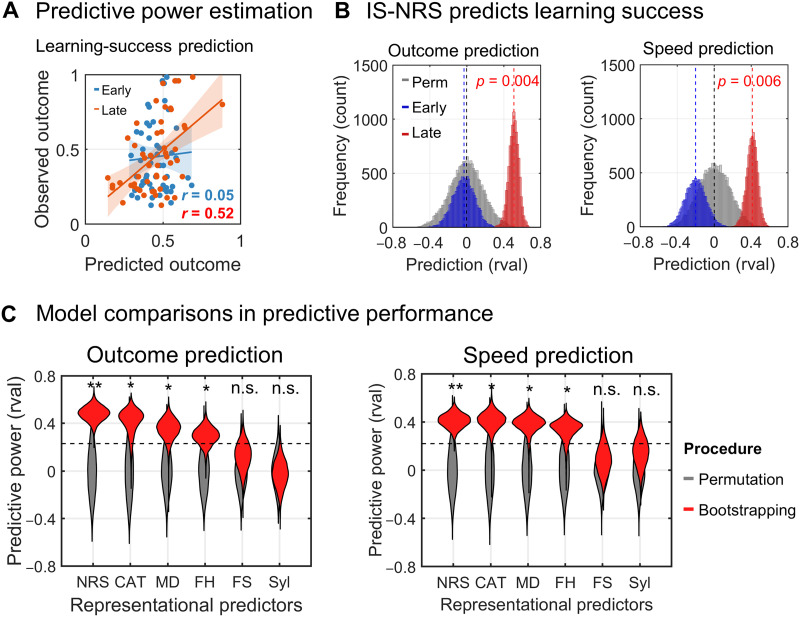
Predictive powers of the IS-NRS and other five model-based representational predictors. (A) Predictive powers were estimated based on the linear correlations between the predicted and observed learning scores. A representative scatter plot with linear fits showed strong predictive power in the late training phase instead of the early. (B) IS-NRS predictive power distributions for the outcome and speed predictions for the early and late phases of training, respectively. Bootstrapping-based distributions were compared with the permutation-based (i.e., Perm) distributions to determine the statistical significance of the prediction models. Models only in the late phase revealed significant effects for both outcome and speed predictions. (C) The IS-NRS showed more predictive power and prediction stability compared with the other five representational predictors. The dashed line indicates the 95th percentile of a permutation-based distribution. Representational predictors: NRS, native listeners’ regional neural model (i.e., IS-NRS); CAT, tone-category model; MD, multidimensional pitch model; FH, F0 height; FS, F0 slope; Syl, syllable-identity model; permutation-based significance test: **, *p* < 0.01; *, *p* < 0.05; n.s., nonsignificant.

We used IS-NRS as an indicator of learners’ nativeness in neural representations of speech sounds. The ROI-based IS-NRS and other model RSA measures were used as candidate predictive features for learning-success prediction analyses (see Figure S4 for the analysis pipeline). We used CV and non-parametric permutation procedures with 10,000 iterations to determine the statistical significance of each predictive model (see Figure 2B for the CV procedure). We also employed the bootstrapping procedure to evaluate the reliability of the prediction models. We found that the IS-NRS in the late phase of training was significantly predictive of learning outcome (permutation test: *p* = 0.004) and speed (permutation test: *p* = 0.006; see Figure 3A and Figure 3B for the predictive powers), whereas the predictive powers were at chance levels for both outcome (*p* = 0.582) and speed (*p* = 0.915) predictions in the early phase (blue-color distributions in Figure 3B).

We conducted additional prediction analyses with fine-tune distinction between different blocks of training. To increase the signal-to-noise ratios of the activation estimation for individual stimulus items, we combined data from two consecutive blocks. Therefore, the whole training session was divided into five parts (i.e., blocks 1–2, 2–3, 3–4, 4–5, and 5–6). We recalculated the IS-NRSs for these blocks and reconducted the learning-outcome and -speed prediction analyses. The results are shown in Figure S7. We found that prediction powers increased as a function of training blocks. Only the IS-NRSs at the last three blocks (i.e., blocks 4–5 and blocks 5–6) were predictive of learning success. These additional results were consistent with the above prediction results showing that the IS-NRSs at the initial phase of training were not predictive of the learning speed and outcome. Altogether, these results demonstrate that the degree of nativeness of the neural representational structure in the late training sessions is tightly related to individual differences in learning efficacy.

To further compare the predictive power of IS-NRS with other model-based representational measures, we conducted the same predictive modeling with other model-based RSA measures as predictors. Four tone-category-related RDMs (i.e., CAT, MD, F0 height, and F0 slope) and one segmental-unit-related RDM (i.e., Syllable) were used to generate RSA representational measures for all ROIs (Figure S4A). With the predictive modeling, we found that the IS-NRS yielded the highest predictive power (median *r*_[predicted,observed]_ = 0.510, *p* = 0.004 for outcome prediction; median *r*_[predicted,observed]_ = 0.412, *p* = 0.006 for speed prediction). Three of the tone-category-related RDMs also yielded predictive powers significantly better than chance (*CAT*: outcome prediction: *r*_[predicted,observed]_ = 0.430, *p* = 0.013, *SD* = 0.097; speed prediction: *r*_[predicted,observed]_ = 0.416, *p* = 0.012, *SD* = 0.077; *MD*: outcome prediction: *r*_[predicted,observed]_ = 0.379, *p* = 0.014, *SD* = 0.082; speed prediction: *r*_[predicted,observed]_ = 0.391, *p* = 0.013, *SD* = 0.074; *F0 height*: outcome prediction: *r*_[predicted,observed]_ = 0.304, *p* = 0.025, *SD* = 0.072; speed prediction: *r*_[predicted,observed]_ = 0.353, *p* = 0.017, *SD* = 0.083). However, the F0 slope and Syllable models did not show significant better-than-chance predictive powers (*F0 slope*: outcome prediction: *r*_[predicted,observed]_ = 0.036, *p* = 0.388, *SD* = 0.119; speed prediction: *r*_[predicted,observed]_ = 0.083, *p* = 0.273, *SD* = 0.118; *Syl*: outcome prediction: *r*_[predicted,observed]_ = −0.053, *p* = 0.649, *SD* = 0.120; speed prediction: *r*_[predicted,observed]_ = 0.151, *p* = 0.182, *SD* = 0.118).

To further confirm that the predictive power of the IS-NRS was not due to sharing the same segmental information (i.e., consonants and vowels) between learners and native listeners, we recalculated the IS-NRS while additionally controlling for the Syl model. We confirmed that the resulting predictive power remained significant (outcome prediction: *r*_[predicted,observed]_ = 0.510, *p* = 0.003; speed prediction: *r*_[predicted,observed]_ = 0.403, *p* = 0.008). We also examined to what extent the predictive power of the IS-NRS was due to the joint variances of F0 height and slope representations by controlling for the variance of the two RDMs. We found that the predictive power of the IS-NRS was diminished (outcome prediction: *r*_[predicted,observed]_ = −0.056, *p* = 0.669; speed prediction: *r*_[predicted,observed]_ = −0.081, *p* = 0.732) when controlled for the variances of both RDMs. These results indicate that the representational models derived from native listeners’ neural patterns and the resulting IS-NRSs outperform other representational measures in differentiating successful from less successful learners.

To examine whether the ROIs with positive or negative correlation patterns contributed equally to the predictive performance, we reran the predictive modeling with two feature selection procedures to disentangle the effects of the two types of ROIs. In one feature selection procedure, we only selected ROIs that showed positive correlations between the IS-NRS and learning performance in the training sets to build predictive models, while in another feature selection procedure, we only selected the ROIs with negative correlations. We found that those models with only positive-correlation ROIs were able to significantly predict learning success while the predictive powers with negative-correlation ROIs were at chance. These results indicate that more native-similar neural representations for the learners are associated with higher learning efficacy.

**Figure 4.**
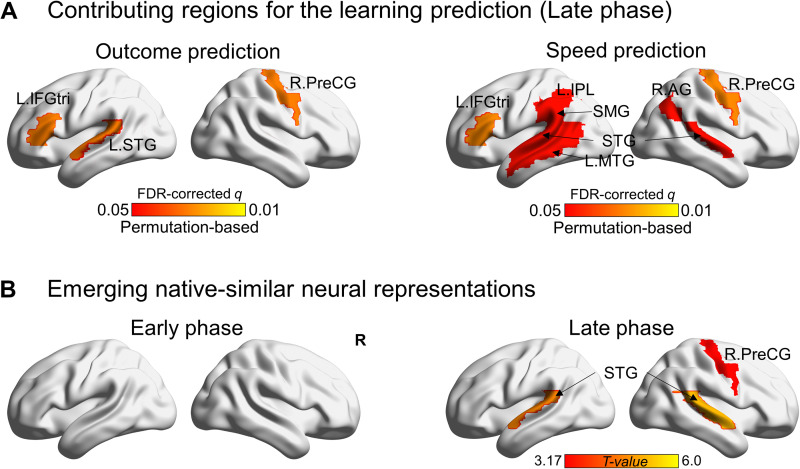
The brain regions that significantly contributed to the predictive models and regions that showed significantly emerging native-similar neural representations. (A) Regions significantly contributing to learning outcome and speed predictions in the late phase of training. Permutation-based FDR-corrected *q* = 0.05. (B) Brain regions that showed emerging native-similar neural representations in the late phase of training. FDR-corrected *q* = 0.05. ROI abbreviations: L.IFGtri, triangular part of left inferior frontal gyrus; L.IPL, left inferior parietal lobe; L.MTG, left middle temporal gyrus; STG, superior temporal gyrus; SMG, supramarginal gyrus; R.AG, right angular gyrus; R.PreCG, right precentral gyrus; L, left hemisphere; R, right hemisphere.

To identify brain regions that significantly contributed to the prediction models with IS-NRS, we estimated the statistical significance of each region using a non-parametric permutation-based approach. In the predictive modeling, we generated a bootstrapping-based correlation distribution and a permutation-based null distribution (10,000 iterations) for each region based on the training sets (i.e., 90% of randomly selected learners, *n* = 48). The median of the bootstrapping distribution for a given region was compared with the 95th percentile of its corresponding null distribution to determine statistical significance. Multiple comparison correction was conducted based on the FDR approach. We found that a speech-related brain network, including the triangular part of left inferior frontal gyrus (L.IFGtri), left inferior parietal lobule (L.IPL), left supramarginal gyrus (L.SMG), bilateral STG, left middle temporal gyrus (L.MTG), right angular gyrus (R.AG), and R.PreCG showed significant contribution to the speed-prediction modeling in the late phase (Figure 4A, right panel). Similarly, L.IFGtri, L.STG, and R.PreCG contributed significantly to the outcome prediction (Figure 4A, left panel).

Additional searchlight IS-NRS analyses within the bilateral STG were conducted to identify which STG subregions contributed to individual differences in learning success. The IS-NRSs were significantly correlated with learning outcomes primarily in the middle and anterior portions of the STG (see Figure S8). Taken together, these results indicate that learners with greater IS-NRS (i.e., more nativeness in neural representations) in the fronto-temporoparietal speech perception network are more successful in learning to categorize novel speech categories.

To further examine whether individual differences in the neural representations at the initial phase of training relate to the individual differences in the neural representations at the late phase, we conducted additional prediction analyses with six neural representational measures (i.e., IS-NRS [native-similar representations], CAT [tone category representations], F0 height [pitch height representations], F0 slope [pitch direction representations], MD [multidimensional pitch representations], and Syl [syllable representations]) as predictive features derived from the first two blocks to predict these representational measures at the last two blocks. To increase the signal-to-noise ratio of the nRDMs, we combined the data from blocks 1 and 2 as well as blocks 5 and 6, respectively. Across the whole brain (94 ROIs), we did not find any region showing a significant prediction effect after correction (i.e., FDR *q* = 0.05). This finding suggests that the initial neural representations may change significantly following training, with successful learners’ representations having native-similarity relative to less successful learners.

### Multidimensionality in Learners’ Neural Representations Contributes to the Learning Success

**Figure 5.**
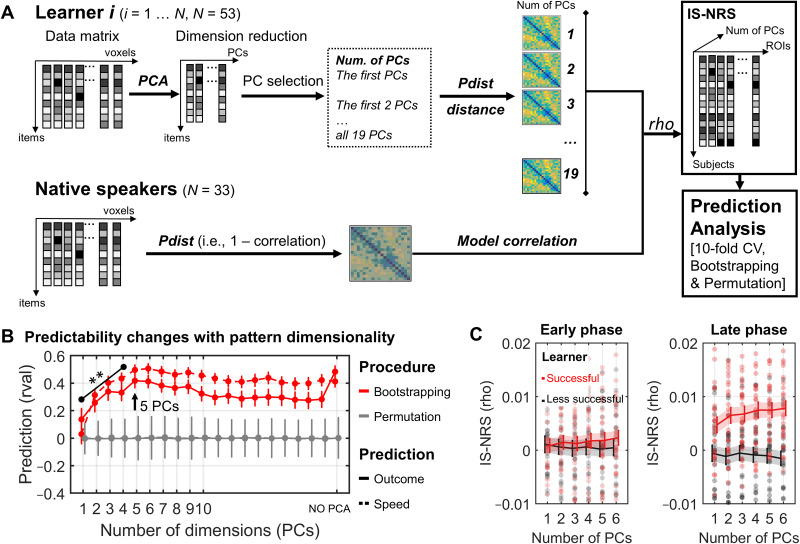
Moderate-to-high dimensionality of learners’ native-similar neural representations best predicts individual learning success. (A) The IS-NRS was recalculated with a dimensional decomposition procedure in which learners’ activation patterns were decomposed into principal components (PCs). We constructed learners’ nRDMs with different numbers of PCs (from 1 to *p*, *p* = number of PCs). The non-native learners’ dimension-constrained nRDMs were then correlated with native nRDM individually to calculate IS-NRS. These IS-NRSs deriving from different numbers of PCs were then used to predict learning success. (B) Predictive power reached a plateau with around five PCs (the black arrow). Predictive powers increased as a nonlinear function of dimensionality. ** *p* < 0.01. (C) Group differences in IS-NRS across training phases. Learners were split into two groups, successful and less successful, based on the median of their outcomes. In the late phase of training, successful learners show more robust native-similar neural representations (i.e., IS-NRS) compared to less successful learners. This group difference was more salient in the moderate-to-high dimensional space than that in the low-dimensional space.

To further reveal the nature of the dimensionality of the emerging native-similar neural representational structure underlying successful learning, we used PCA with the singular value decomposition algorithm to decompose learners’ brain activation patterns of the stimuli into independent PCs and recalculated the IS-NRS with PC-constrained nRDM for predictive modeling (see Figure 5A for graphical analysis procedure). This procedure allows us to assess how many dimensions of the learners’ representations underlie individual differences in learning success. We found that dimensionality significantly modulated the predictive powers for both learning-speed and -outcome predictions. Predictive power increased as the dimensionality increased. Importantly, predictive powers reached a plateau with approximately five PCs (speed prediction: median *r* = 0.52, *p* = 0.001; outcome prediction: *r* = 0.42, *p* = 0.005; 1PC’s vs. 5PCs’ predictive power: *p*’s < 0.001; Figure 5B).

We also conducted the same prediction analysis with PCA for six predefined speech-perception-related regions and found that the number of PCs for the maximum predictive powers were varied across regions (ranging from two to nine PCs; see Figure S9). A more straightforward demonstration is shown in Figure 5C. We extracted the IS-NRSs (controlled for both hand-response and Syllable RDMs) from the significant contributing regions (i.e., L.IFGtri, L.STG, and R.PreCG) and compared the IS-NRSs between the successful and less-successful learners across different numbers of PCs. Two groups of learners were created based on the median split of the learning outcome (successful: *n* = 26, *M* = 65.0%; less successful: *n* = 25, *M* = 24.4%; two of them in the median line were removed). We found that successful learners showed greater IS-NRS than less successful learners (group-by-dimensionality ANOVA; main effect of the group: *F*(1, 49) = 16.33, *p* < 0.001) but only in the late phase; while the group differences significantly increased in the dimensionality, and group difference effect reached the maximum at 5–6 PCs, which was evidenced by a significant group-by-dimensionality interaction effect (*F*(5, 245) = 4.84, *p* < 0.001). These results indicate that successful learners use a multidimensional but also cost-efficient neural representational mechanism (i.e., a moderate number of dimensions) to encode the newly-acquired speech categories.

### Neural Sensitivity to Feedback-Valence in the Frontostriatal System Contributes to Individual Differences in Learning Success and in the Nativeness of Neural Representations

**Figure 6.**
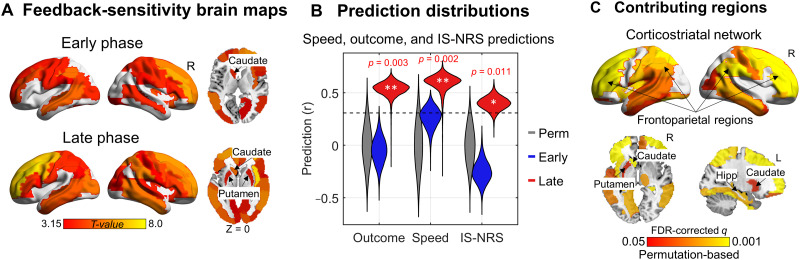
Neural sensitivity to feedback valence predicts individual learning success and the degree of nativeness in neural representational structure in the late phase of training. (A) Feedback-valence sensitivity brain maps for both early and late phases of training. Feedback-valence sensitivity was measured by the ROI-based multivariate feedback-type classification analysis. The group-level classification maps were thresholded at FDR-corrected *q* = 0.05. (B) Violin graphs show the prediction distributions of predicting speed and outcome as well as the IS-NRS of L.IFGtri, bilateral STG, and R.PreCG. Neural feedback sensitivity in the late phase significantly predicted behavioral learning success and the robustness of learners’ native-similar neural representations (i.e., IS-NRS). The dashed line indicates the 95th percentile of a permutation-based distribution. (C) Corticostriatal regions significantly contributed to the learning-outcome prediction (see Figure S10 for those significantly contributing regions to learning-speed and IS-NRS predictions). The color bar indicates the significance (vs. permutation distributions) of the ROIs in correlating the neural feedback sensitivity with the learning outcome, derived from the feature selection and permutation procedures. Permutation-based FDR-corrected *q* = 0.05. ROI abbreviations: Hipp, hippocampus; L, left hemisphere; R, right hemisphere.

To evaluate the extent to which the neural sensitivity to feedback valence is a driving factor of the behavioral learning success (i.e., outcome and speed) and the degree of nativeness of neural representational structure (i.e., IS-NRS), we used the multivariate feedback-type classification accuracy as a neural feedback-sensitivity index to predict the learning performance and IS-NRS. Higher feedback-type classification accuracy indicates more sensitivity to feedback valance (i.e., more robust feedback-valance representations) in the brain. At the group level, with a CV procedure strictly balancing the number of correct and incorrect feedback trials, we found that widespread brain regions showed significantly above-chance classification accuracy for both early and late phases of training (Figure 6A), including cortical and sub-cortical striatal areas. Note that, quantitatively, the classification accuracies in the late phase were slightly higher than in the early phase, especially in the frontostriatal regions.

The most significant feedback-sensitive regions across training phases were within the frontostriatal network, which is consistent with previous findings derived by univariate activation analysis that used contrasts of correct vs. incorrect feedback (Feng et al., 2019; Yi et al., 2016). Importantly, the neural feedback sensitivity in the late phase significantly predicted learners’ behavioral learning outcome (median *r*_[predicted,observed]_ = 0.54, *p* = 0.003; permutation test) and speed (median *r*_[predicted,observed]_ = 0.60, *p* = 0.002) (Figure 6B). In contrast, the predictions with feedback classification accuracies in the early phase were not significantly better than chance (*p*’s > 0.05). Furthermore, the neural feedback sensitivity in the late phase significantly predicted the IS-NRSs (median *r*_[predicted,observed]_ = 0.40, *p* = 0.011) of the L.IFGtri, bilateral STG, and R.PreCG (IS-NRSs collapsed across these regions; see Figure 6B), where these regions showed significant predictive powers of learning success as well as the emergent native-similar neural representations in the late phase relative to the early phase.

The feedback-sensitive regions that significantly contribute to the learning predictions were identified in the frontostriatal network (Figure 6C, outcome prediction regions; also see Figure S10 for regions significantly contributing to the speed and IS-NRS predictions), which indicates that the neural sensitivity of feedback valence at the late phase of training within this network is a neuromarker of tone-category learning success. The most contributing regions in predicting the IS-NRSs were also within the frontostriatal network, including the L.IFGtri, left caudate, right AG, right IFGorb, right middle frontal gyrus, and right posterior cingulate cortex (permutation-based FDR-corrected *q* < 0.05). These results demonstrate that the frontostriatal network plays an important role in facilitating the formation of native-similar neural representations.

## DISCUSSION

We employed a novel IS-NRS analysis and rigorous predictive modeling approach to examine the neural underpinnings of individual differences in non-native speech category learning success. We demonstrate that native-similar neural representational structure emerges during training, and that the degree of nativeness of the neural representations in the left IFG, left STG, and right PreCG is robustly predictive of behavioral learning success. The emerging native-similar neural representations in successful learners are multidimensional and economical in encoding pitch-related phonetic/phonological category information. Further, individual differences in neural sensitivity to feedback valence within the frontostriatal network are highly predictive of individual differences in learning success and of the degree of nativeness of the emerging representations. These findings provide new insights into the neural representational mechanism underlying successful non-native speech category learning and the role of feedback in mediating individual differences in learning success.

### The Nativeness in Neural Representational Structure Predicts Sound-to-Category Learning Success

It has been previously demonstrated that task-general and acoustic-invariant neural representations of Mandarin tone categories for native listeners are evidenced in the superior temporal areas and IPL using multivariate pattern classification (Feng, Gan et al., 2018; Feng et al., 2021). While this classification approach reveals category-level representations, this analytic method cannot capture the fine representational structures underlying the neural activation patterns. Here we used native listeners’ nRDMS as a native representation model to estimate learners’ neural representational structure and assess the extent to which native-similar representations emerged during learning at the group level and in relation to individual differences in learning success. At the group level, the native-similar neural representational structure emerged in the late phase of training, similar to the emerging neural representations of tone categories and multidimensional pitch information (see Figure S5), which suggests that for adult learners, feedback-based training protocol could not only facilitate the formation of task-relevant categorical representations (Chandrasekaran, Koslov, & Maddox 2014; Feng et al., 2019) but also result in representational structures that were increasingly similar to native listeners within just hundreds of training trials. This finding is consistent with the previous observation that neural representations of tone category emerge following training (Feng et al., 2019), and further reveals the native-similar nature of the representational structure underlying successful sound-to-category acquisition.

In addition to the emerging native-similar representations, a key finding is that greater neural similarity in representational structure between learners and native listeners (i.e., IS-NRS) predicts better learning performance. This finding suggests that IS-NRS is a robust neural representational indicator of sound-to-category learning success. The prediction results are validated by the rigorous predictive modeling approach with CV, bootstrapping, and permutation procedures, and are not explained by the contextual factors, response similarity, or individual differences in tone identification performance in the first block of training. The left IFG, STG, and right PreCG are crucial brain regions that reliably contributed to the learning-success prediction. These findings demonstrate that more successful learners reveal greater similarity to native listeners in their neural representations of Mandarin tone categories, even though the mechanisms underlying how the category representations are acquired may be fundamentally different (e.g., unsupervised vs. feedback-based learning) (Hernandez et al., 2005; Lim et al., 2019; MacWhinney, 1998).

The native listeners’ neural representational dissimilarity structure serves as an excellent tone-contrast model to quantitatively evaluate the degree of nativeness of neural representational patterns for the learners. The native listeners’ dissimilarity structure is also better at differentiating successful from less successful learners compared to other representational models (i.e., CAT, MD, etc.). The IS-NRS prediction model yielded the best prediction powers for how fast and how well learning could be achieved among other predefined pitch-related category models, reflecting the predictive accuracy as well as the reliability of the predictive models revealed by the bootstrapping procedure (Figure 3C). Previous studies have documented several neural indicators of speech learning success (Deng et al., 2016; Golestani & Zatorre, 2009; R. Liu & Holt, 2011; Myers & Swan, 2012; Sheppard et al., 2012; Wong & Ettlinger, 2011; Wong et al., 2007; Wong et al., 2011; Zhang et al., 2009). However, these studies have largely focused on pre-training neural measures to predict learning outcomes or on examining the group-level neural changes in response to training. Here, we categorically focus on the neural representational dynamics during the process of learning and on how neural plasticity contributes to individual differences in learning. Our results provide key insights into how successful learners form multidimensional and economical representations as a function of training with a goal of more efficient categorization.

### The Dimensionality of the Emerging Native-Similar Neural Representational Patterns

Theoretical models in L2 acquisition, largely in the domains of grammar and syntax, posit that the representational structure in L2 learners may be shallow and inefficient (Clahsen & Felser, 2006a, 2018). However, in terms of non-native speech category learning, our results demonstrate that the emerging neural representations of newly acquired speech categories for successful learners are not only significantly similar to those of native listeners but also multidimensional and cost-efficient, where the speech categories are encoded in a neural representational space with a moderate number of dimensions. Mathematically, a high-dimensional representational space provides flexibility in encoding different categories but may come with a greater cost in terms of neural resources. In contrast, a low-dimensional space expends fewer resources but may not be capable of robustly differentiating behaviorally relevant categories. An optimal learning-induced representational mechanism would need to balance these two competing factors—maximizing behaviorally relevant information in the signal with minimal resources to encode information (Gervain & Geffen, 2019; Tang et al., 2019).

Using the single vector decomposition approach, we decomposed the neural patterns of speech sounds into independent dimensions and reconstructed the representational spaces parametrically with different numbers of dimensions to assess the relationship between dimensionality and predictive power as well as to estimate how many dimensions are needed to differentiate successful learners from less successful learners. Our results showed that predictive powers changed as a nonlinear function of dimensionality, which reflects an interaction between learning success and dimensionality. Successful learners’ neural representations showed increasingly native similarity as the number of dimensions increased, whereas less successful learners did not show such a relationship. Importantly, learning-success predictions did not increase linearly with the number of dimensions increase (i.e., close to plateau at around five dimensions). This result suggests that the emerging neural representations in successful learners are cost-efficient, in which activation patterns encode the new categories with a limited number of dimensions that can maximally differentiate them, similar to native listeners (Gandour, 1983; Gandour & Harshman, 1978). Using other representational models’ RSA measures as predictors, we further demonstrate that multidimensional pitch information is a critical constituent of the emerging native-similar neural representations for successful learners. Consistent with previous findings in native listeners (Chandrasekaran, Gandour, & Krishnan, 2007; Gandour & Harshman, 1978), we posit that pitch height and direction (i.e., contour) are important category-defining components that represent tone-category distinctions in successful learners. Although prior behavioral studies have shown that other dimensions may also differentiate tones (Gandour & Harshman, 1978), we found that when we controlled for the variance of both F0 height and slope, the prediction was diminished. These results suggest that pitch height and direction are two critical components underlying both the native listeners’ and successful learners’ neural representations, in line with our original hypothesis.

The brain areas that significantly contributed to the learning-success prediction are within a large speech-related network involved in encoding pitch information for both native listeners and successful learners. These include the left IFG, left IPL, bilateral STG, left SMG, left MTG, right AG, and PreCG. Intriguingly, these brain areas encode the two pitch components differently for learners at the group-level. The bilateral STG, PostCG, PreCG, and the left IFG are dominated by F0 height, whereas many fewer regions are dominated by representing F0 slope (see Figure S5). However, for native listeners, the above regions encode the multidimensional pitch information of the categorical representation. It is important to note that sound-to-category training only involved 240 trials. The mean accuracy for even the successful learners (*n* = 26) in the last training block (*M* = 70%) is therefore far from perfect, compared to the native speakers (*M* = 97%). Therefore, the greater dominance of pitch height in learners relative to native listeners may be because the learners are still novices. In line with a recent study demonstrating changes at early auditory processing stages with extensive multi-day sound-to-category training (Reetzke et al., 2018), we posit that a more extended training phase may yield better neural alignment of dimensional structure between native listeners and successful learners.

### Neural Sensitivity to Feedback Valence Drives Learning Success and the Emergence of Native-Similar Neural Representations

Our results demonstrate the significant similarity between native listeners and successful non-native learners in how tone-category-related information is represented in the brain. It is important to note that this significant neural similarity emerges following a relatively short period of sound-to-category training, which fundamentally differs from the mechanisms underlying category acquisition during infancy. Acquiring speech categories in adulthood is argued to require greater supervision, and recent models (e.g., the dual learning systems model) have highlighted the role of multiple corticostriatal systems in mediating adult speech learning (Chandrasekaran, Koslov, & Maddox, 2014; Chandrasekaran, Yi, & Maddox, 2014; Maddox & Chandrasekaran, 2014). Here, we provide supporting evidence from the perspective of individual differences in learning success that the neural sensitivity to feedback valence in the frontostriatal system is highly predictive of both behavioral learning success and the emerging native-similar neural representations. We posit that learners are reliant on feedback processing to update the internal representation, which could guide the formation of correct sound-to-category representations and efficient categorization behaviors.

Individual differences in feedback processing and sensitivity are presumably critical factors associated with individual differences in learning outcomes. A previous study has identified the putamen, a core region in the striatum, dynamically coupling with the representational areas in the left STG when learners are processing corrective feedbacks (Feng et al., 2019). In expanding this finding with a novel prediction analytic method, we found that individual differences in neural feedback sensitivity in a more extended cortico-striatal network, including the striatum as well as lateral and medial frontal, precentral gyrus, inferior parietal cortex, and hippocampus areas robustly contributed to the prediction of learning success and the degree of native-similar representations. These findings suggest that feedback sensitivities in the two putative category learning systems (i.e., reflective and reflexive systems) are critical neural sources mediating individual differences in speech category learning success, at least during the transition learning stage (from novice to experienced phase).

The neural sensitivity to feedback valence is prominent in the late training phase relative to the early phase. Similarly, the representation/learning-success predictions (based on feedback-valence sensitivity) are more powerful for the late relative to the early training phase. We posit that trial-by-trial corrective feedback information facilitates rapidly updating learners’ internal representations to enhance categorization success. More successful and faster learners likely leverage the feedback better, leading to the more native-similar multidimensional representations of the acquired speech categories. During sound-to-category learning, interactions between the striatum, auditory cortex, and frontoparietal regions might enable the integration of perceptual representation and feedback valence, mediating the shift from novice to skilled behavioral performance (Reetzke et al., 2018).

Learning non-native novel phonemic contrasts is a key step toward acquiring new words in a foreign language. Previous studies have demonstrated that both learning non-native phonemic contrasts and learning new words rely on the feedback/reward-sensitive striatal regions (Feng et al., 2019; Lim et al., 2019; Ripolles et al., 2014, 2016) and interactions within corticocortical and corticostriatal networks (Li et al., 2014; Lopez-Barroso et al., 2013; Shtyrov, 2012). The striatal activations are associated with domain-general reward processes, where a reward signal (e.g., gaining money or receiving feedback) may facilitate the formation of new memories in general (Adcock et al., 2006; Wolosin et al., 2012) and drive the acquisition of different language components (beyond phonetic/phonological learning). The interaction between the striatum and cortical regions has been proposed to be a neural driving force for the formation of cortical representations in language learning (Feng et al., 2019; Ripolles et al., 2014). Here, we further demonstrate that individual differences in learning success and the robustness of the emerging native-similar neural representations are both associated with the feedback sensitivity in the corticostriatal network. This corticostriatal interaction mechanism may not be restricted to the learning of novel non-native phonemic contrasts but also could be used in other aspects of language learning (e.g., learning new words and grammar). Further studies need to be conducted across different domains of language learning to directly address this question.

To what extent can our results generalize to typical language learning contexts? Prior studies have trained participants on a sound-to-meaning training paradigm that involves learning novel words as well as tone categories (Deng et al., 2016; Wong et al., 2007). Similar to the current study, large individual differences underlie learning performance in this learning context as well. Interestingly, during the initial word learning, learners often make lexical errors, but by the end of the training, most errors are in disambiguating tonal categories. Indeed, a prior study demonstrated that learning success in such a paradigm may be driven by poorer representations of tone category information in subcortical auditory regions (Chandrasekaran et al., 2012). Thus, representational plasticity may underlie individual differences in learning to map pitch information irrespective of learning context. However, it is also possible that in a more ecological word learning context, there would be a need for greater couplings among the lexical-semantic network, STG, and the reward-related corticostriatal pathways. In this context, individual differences in learning success may depend on emerging representations of tone categories as well as lexical-semantic representations.

### Conclusion

Using the multivariate intersubject RSA and predictive modeling approaches, we deconstructed the neural sources of interindividual differences in learning success during the process of learning to map non-native speech sounds into discrete categories. Successful learners can build robust and detailed speech representations that are similar to those in native listeners. The greater similarity between non-native learners and native listeners in neural representations of tone-category-related pitch information is associated with more rapid learning and better learning outcomes. Neural representations in successful learners are encoded in a cost-efficient manner: The representational space is multidimensional but with a limited number of dimensions that maximize the categorization of newly acquired speech categories. The emerging native-similar representations in more successful learners are associated with neural sensitivity to feedback valence in a distributed frontostriatal network. We provide new evidence for the neural mechanisms underlying the successful acquisition of non-native speech categories in adulthood and insights into the scaffolding for the development of individualized speech training protocols that maximize learning outcomes with effective feedback.

## ACKNOWLEDGMENTS

This work was supported by grants from the General Research Fund (Ref. No. 14619518 to Gangyi Feng) of the Research Grants Council of Hong Kong, Direct Grant for Research (Ref. No. 4051137 to Gangyi Feng) by The Chinese University of Hong Kong, and the National Institute on Deafness and other Communication Disorders of the National Institutes of Health (Award No. R01DC013315 to Bharath Chandrasekaran). We thank Casey Roark for her helpful comments on an early version of the manuscript.

## FUNDING INFORMATION

Gangyi Feng, General Research Fund of the Research Grants Council of Hong Kong, Award ID: 14619518. Gangyi Feng, Direct Grant for Research by The Chinese University of Hong Kong, Award ID: 4051137. Bharath Chandrasekaran, National Institute on Deafness and Other Communication Disorders (https://dx.doi.org/10.13039/100000055), Award ID: R01DC013315.

## AUTHOR CONTRIBUTIONS

**Gangyi Feng**: Conceptualization; Data curation; Methodology; Software; Formal analysis; Investigation; Resources; Writing – Original Draft; Writing – Review & Editing; Visualization; Supervision; Project administration; Funding acquisition. **Yu Li**: Investigation. **Shen-Mou Hsu**: Investigation. **Patrick C. M. Wong**: Resources; Writing – Review & Editing; Supervision. **Tai-Li Chou**: Resources; Project administration. **Bharath Chandrasekaran**: Resources; Supervision; Writing – Original Draft; Writing – Review & Editing; Funding acquisition.

## COMPETING INTERESTS

Patrick C. M. Wong is a founder of a company in Hong Kong supported by a Hong Kong SAR government startup scheme for universities.

## TECHNICAL TERMS

Multivariate representation-based neural mechanisms: Refers to neural mechanisms underlying information representation revealed by multivariate pattern analyses.
Corticostriatal learning systems: Refers to a set of cortical and striatal regions that contribute to learning elements of a new language (e.g., new speech categories).
Intersubject neural representational similarity (IS-NRS): A neural measure that estimates pattern similarities in neural representational structure between two subjects.
Predictive modeling: A modeling process wherein an algorithm is trained to model the relationship between an outcome and a set of predictor variables, and validated with unseen data.
Cross-validation: A model validation technique used to evaluate the generalization performance of a machine learning model to an independent dataset.
