# Supplementary material for: Emerging Native-Similar Neural Representations Underlie Non-Native Speech Category Learning Success
Source: Neurobiol Lang (Camb). 2021 Jun 9;2(2):280–307. doi: 10.1162/nol_a_00035 (PMC8345815; doi:10.1162/nol_a_00035)
Supplement: Supplementary file 1 [file nol-2-2-280-s001.pdf]

Supplementary Materials for

Emerging native-similar neural representations underlie non-native speech category learning success

Gangyi Feng*, Yu Li, Shen-Mou Hsu, Patrick C.M. Wong, Tai-Li Chou, Bharath Chandrasekaran*

*Corresponding authors:

GF, Email: g.feng@cuhk.edu.hk; BC, Email: b.chandra@pitt.edu

This PDF file includes:

Figs. S1 to S10

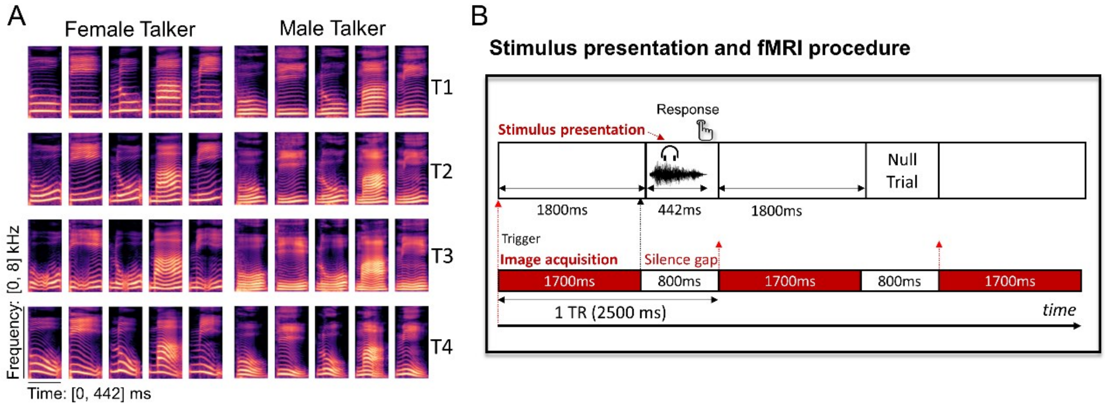

Figure S1. Stimuli and fMRI scanning procedure for native Mandarin speakers. **A**, the spectrogram of sample sound stimuli. The same set of stimuli were used in the tone-category training experiment for learners and tone categorization experiment for native speakers. Each column shows sounds with the same syllable (e.g., /bu/). Each row represents a tone category. T1: high-flat, T2: low-rising, T3: low-dipping; T4: high-falling. **B**, the stimulus presentation and sparse-sampling fMRI scanning procedure for the tone-categorization experiment.

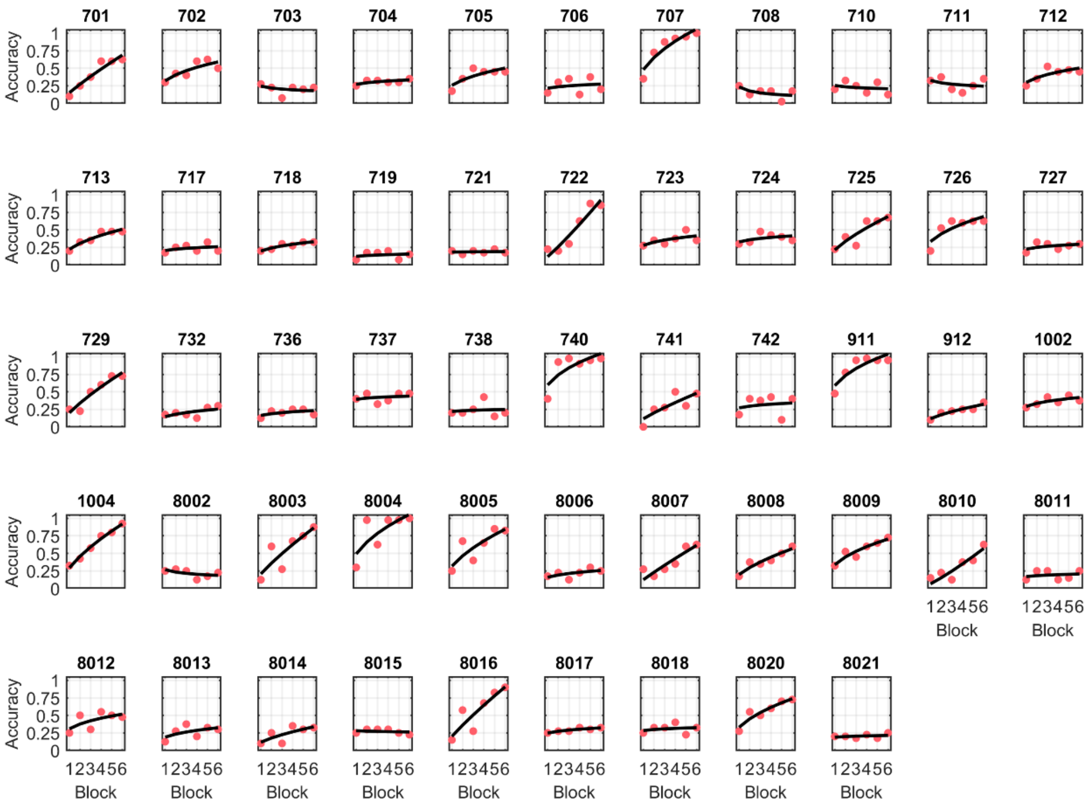

Figure S2. Block-by-block learning performance and learning-curve fitting with a power function for each learner. Red dots = block-by-block tone category identification accuracies. Black lines = model fitting curves. The subject label was plotted at the top of each graph.

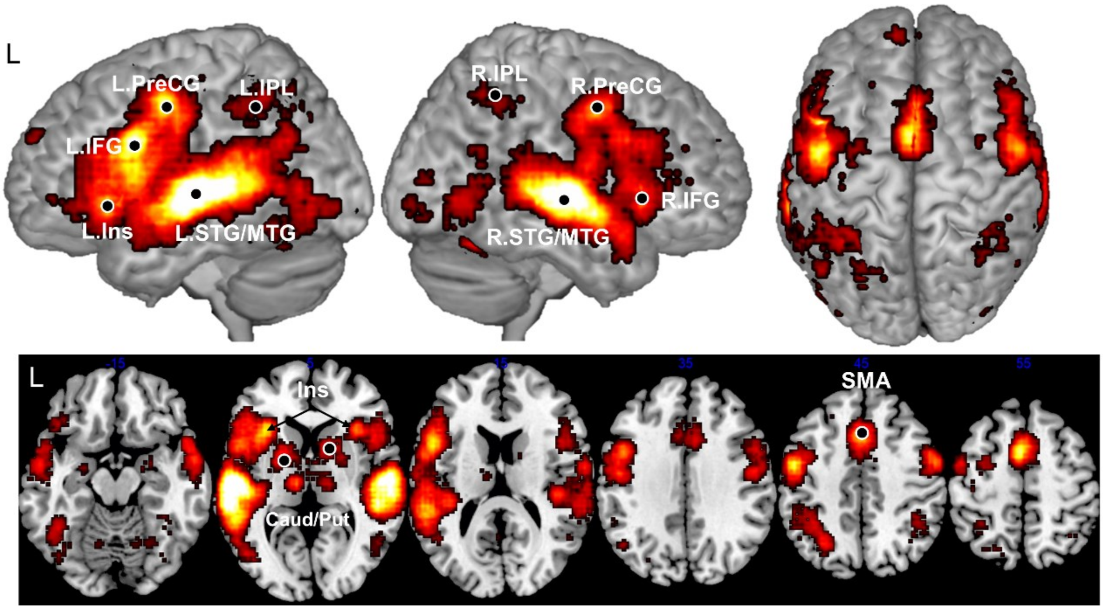

Figure S3. A brain map derived from the Neurosynth meta-analysis was used as a brain mask to extract activation patterns to calculate native neural representational matrices from the native speakers for the behavioral representational similarity analysis (bRSA, see Figure 1D-F). Key regions related to speech/auditory perception were labeled. The total number of selected voxels is 22920 (voxel size = 2 mm^3). The percentage of selected voxels is 15.7%.

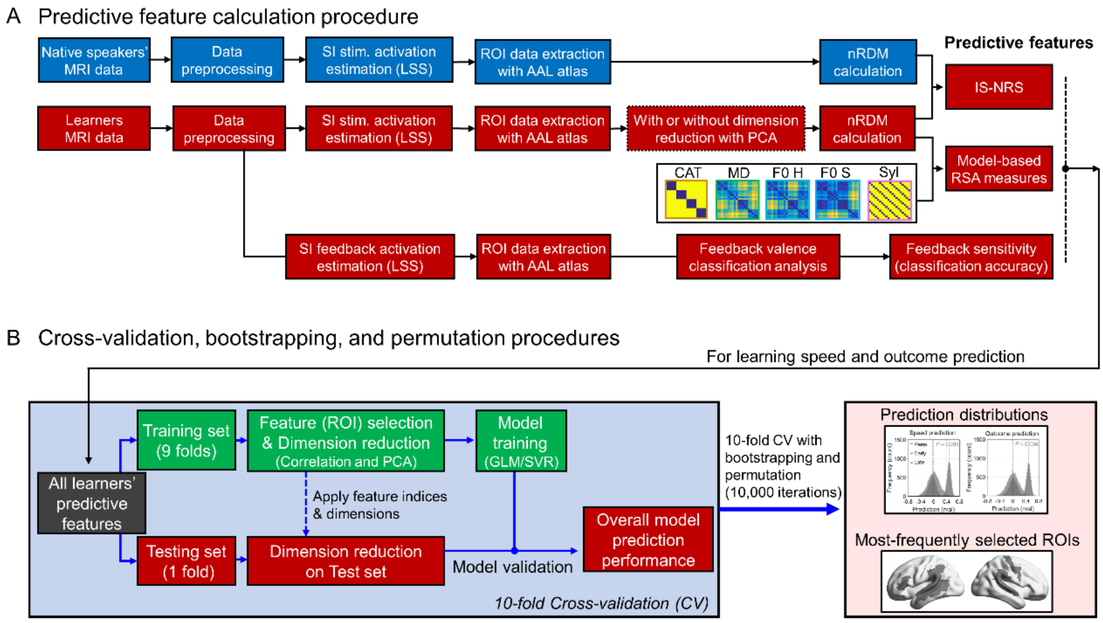

Figure S4. Predictive feature calculation schema and predictive modeling procedures. **A**, predictive feature calculation procedure. Three types of representational predictive features were calculated, including the inter-subject neural representational similarity (IS-NRS), model-based RSA measures (with five model RDMs), and neural feedback sensitivity (i.e., feedback valence classification accuracies). These predictive features were used individually to predict learning speed and outcome. See detailed IS-NRS calculation in the Methods section and Figure 2A and Figure 5A for graphical illustration. **B**, predictive modeling procedure. The 10-fold cross-validation (CV) procedure was used to construct and validate the prediction models. Predictive powers were evaluated by the Pearson correlation between predicted learning scores and the observed scores. Bootstrapping and permutation procedures were employed with the CV procedure to estimate the statistical significance of the prediction models and identify the most contributing regions (i.e., most frequently selected ROIs).

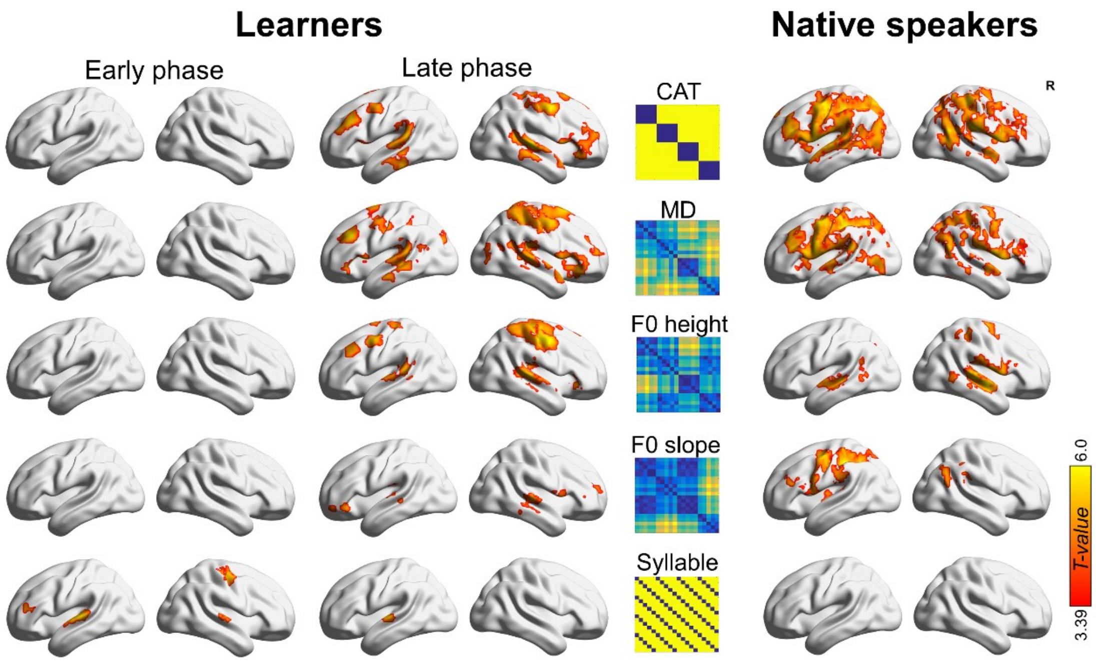

Figure S5. Model-based searchlight RSA brain maps with five predefined RDMs for both non-native learners and native Mandarin speakers. From top to bottom panels: each row displays RSA brain maps for an RDM. Learners' RSA brain maps were calculated for the early and late phases of training separately. For the CAT, MD, and F0 height models, we found spatially similar but less distributed brain patterns between learners (late phase) and the native speakers. For the F0 slope maps, we found that only the bilateral STG and IFG showed significant correlations in the late phase, whereas the native listeners recruited the inferior frontal gyrus, precentral gyrus, and inferior parietal lobule. RSA maps with the Syllable RDM revealed decreasing representations of syllable identity where less significantly correlated regions were found in the late phase compared to the early phase. Brain maps were threshold at the voxel-level $P < 0.001$ and cluster-level FWE-corrected $P < 0.05$.

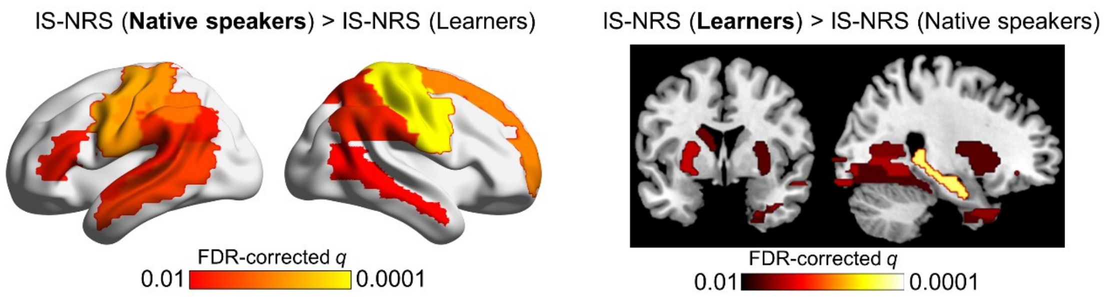

Figure S6. Group comparisons in regional IS-NRS between native speakers and learners. Left panel, regions showed significantly higher IS-NRS for native speakers than that of the learners. Regions shown here were corrected for multiple comparisons with the false discovery rate approach (FDR $q < 0.01$).

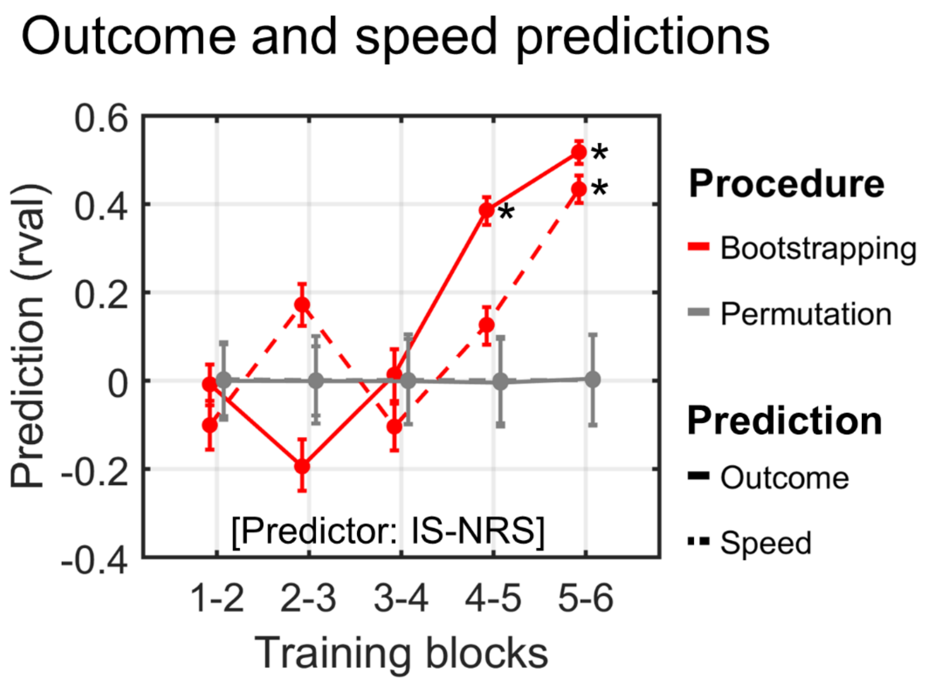

Figure S7. Learning outcome and speed predictability with the IS-NRS as predictive features across training blocks. Brief analysis procedure: single item's brain activations from two consecutive blocks were averaged to increase signal-to-noise ratios. The neural representational dissimilarity matrices were generated for the learners and the regional IS-NRSs were then calculated for prediction analysis (see Methods section for details). Significant learning-success predictions emerged at the last three blocks, which was consistent with the results reported in Figure 3. *, $P < 0.05$, permutation test (i.e., predictions were significantly better than chance). Bootstrapping and permutation procedures were employed with 10,000 iterations. Error bar = quartile; the median of each distribution was shown.

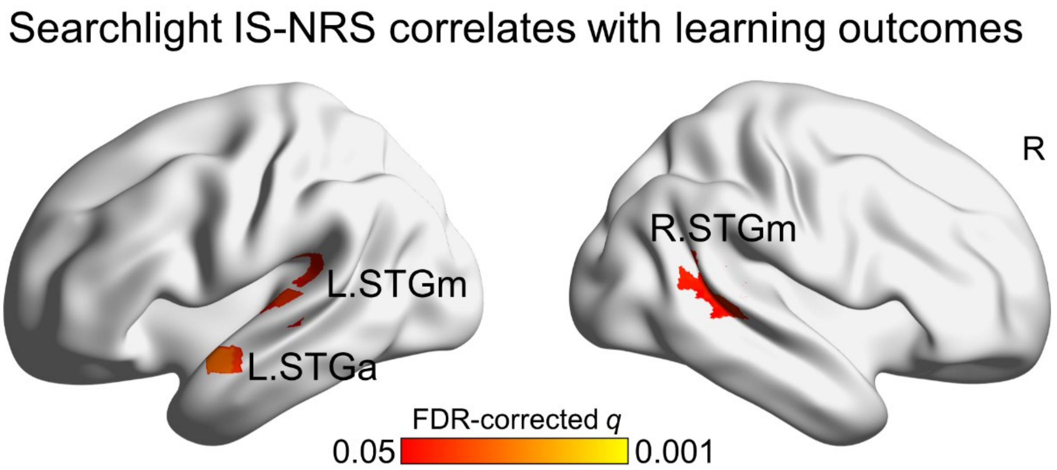

Figure S8. Searchlight-based IS-NRSs correlated with the learning outcome in the middle and anterior portions of the STG. Both learners' and native speakers' brain activation maps were first normalized to standard MNI space. The searchlight analysis was restricted within the bilateral STG defined by the AAL2 atlas. For each searchlight sphere (radius = 3 voxels; ~70 voxels), an average nRDM derived from the native speakers was correlated with each learners' nRDM to calculate IS-NRS. This IS-NRS value was then mapped back to the center of the sphere. This IS-NRS calculation was conducted for each voxel within the bilateral STG to generate IS-NRS maps for each learner. Regression analysis was conducted to examine the relationship between the voxel-wise IS-NRS and learning outcomes.

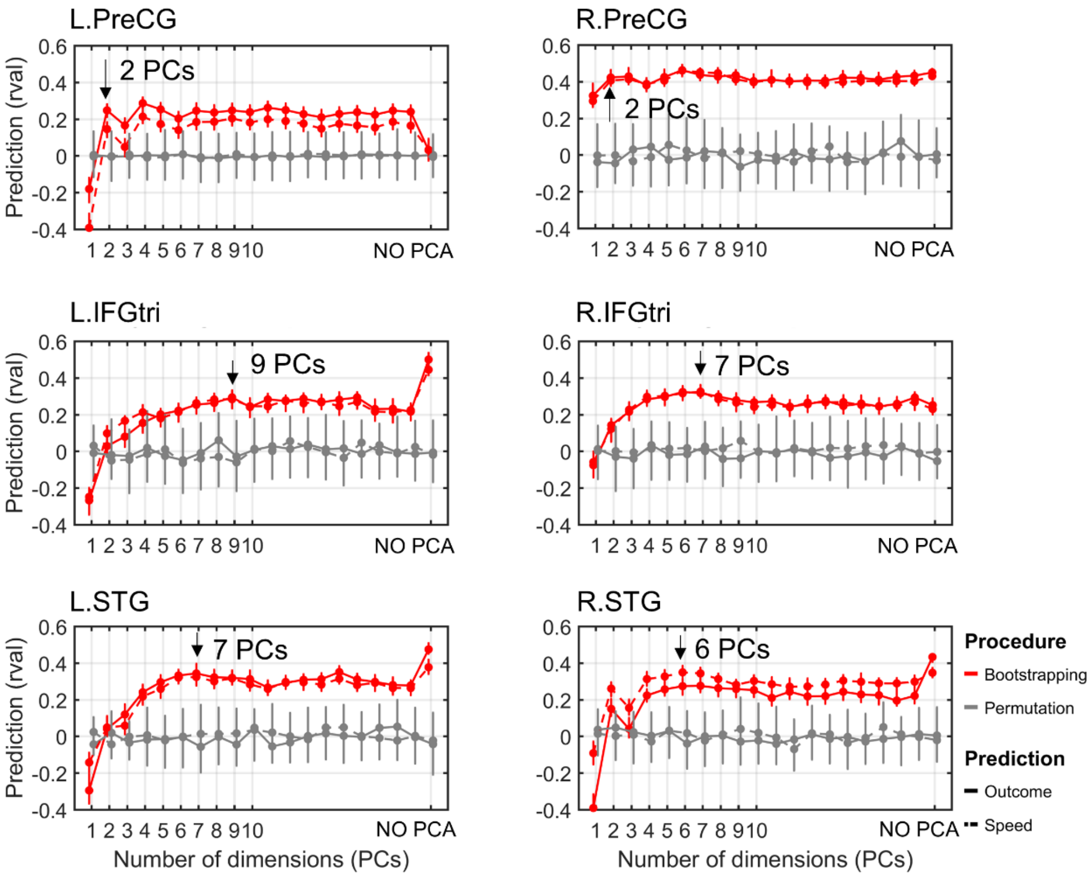

Figure S9. The relationship between the neural pattern dimensionality and learning predictions for six anatomical ROIs. Each cross-validation prediction repeated 1,000 times.

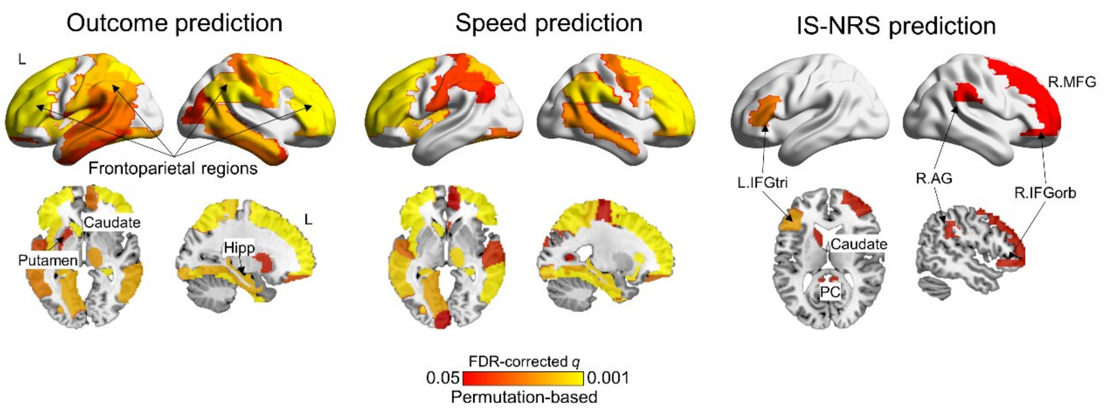

Figure S10. The significant contributing regions in predicting the learning outcome, speed, and IS-NRSs. FDR-corrected $q < 0.05$. Region abbreviation: Hipp, hippocampus; L.IFGtri, triangular portion of the left inferior frontal gyrus; R.AG, right angular gyrus; R.IFGorb, the inferior frontal gyrus pars orbitalis; R.MFG, the right middle frontal gyrus. L, left hemisphere.
